# Domestication may affect the maternal mRNA profile in unfertilized eggs, potentially impacting the embryonic development of Eurasian perch (*Perca fluviatilis*)

**DOI:** 10.1371/journal.pone.0226878

**Published:** 2019-12-31

**Authors:** Tainá Rocha de Almeida, Maud Alix, Aurélie Le Cam, Christophe Klopp, Jérôme Montfort, Lola Toomey, Yannick Ledoré, Julien Bobe, Dominique Chardard, Bérénice Schaerlinger, Pascal Fontaine

**Affiliations:** 1 UR AFPA, University of Lorraine, INRA, Nancy, France; 2 LPGP, UR1037 Fish Physiology and Genomics, INRA, Rennes, France; 3 Sigenae, MIAT, INRA, Castanet-Tolosan, Toulouse, France; Kansas State University, UNITED STATES

## Abstract

Domestication is an evolutionary process during which we expect populations to progressively adapt to an environment controlled by humans. It is accompanied by genetic and presumably epigenetic changes potentially leading to modifications in the transcriptomic profile in various tissues. Reproduction is a key function often affected by this process in numerous species, regardless of the mechanism. The maternal mRNA in fish eggs is crucial for the proper embryogenesis. Our working hypothesis is that modifications of maternal mRNAs may reflect potential genetic and/or epigenetic modifications occurring during domestication and could have consequences during embryogenesis. Consequently, we investigated the trancriptomic profile of unfertilized eggs from two populations of Eurasian perch. These two populations differed by their domestication histories (F1 vs. F7+–at least seven generations of reproduction in captivity) and were genetically differentiated (F_ST_ = 0.1055, p<0.05). A broad follow up of the oogenesis progression failed to show significant differences during oogenesis between populations. However, the F1 population spawned earlier with embryos presenting an overall higher survivorship than those from the F7+ population. The transcriptomic profile of unfertilized eggs showed 358 differentially expressed genes between populations. In conclusion, our data suggests that the domestication process may influence the regulation of the maternal transcripts in fish eggs, which could in turn explain differences of developmental success.

## Introduction

Domestication is an evolutionary and continuous process enabling wild animals to adapt to humans and anthropogenic environments [1,2]. It involves the combination of several genetic and potentially epigenetic modifications driving gene expression and phenotypic changes. Differences in gene expression due to rapid adaptation to new environments were already reported from the first generation of reproduction in captivity in steelhead trout (*Oncorhynchus mykiss*) [3]. Thus, the regulation of genes expression may change early during domestication. The main genetic mechanisms involved are inbreeding, genetic drift and natural, relaxed and artificial selections [1,2]. The two first mechanisms have important consequences when founder populations are small because they rapidly lead to important changes in allelic frequencies [4]. The relaxed selection consists of a reduction of selection pressure on traits which are not necessary anymore in captive conditions. The artificial selection results from the selection of breeders according to phenotypes chosen by humans. Finally, the natural selection occurs and usually eliminates animals which are not adapted to anthropogenic environments [5]. Studies suggest a relationship between epigenetic modifications and phenotypic plasticity in response to the environment in some animal and plant species [6]. In fish, the current knowledge shows a modification of the epigenetic signature in individuals reared in hatcheries in comparison to their wild counterparts in salmonids and the European sea bass (*Dicentrarchus labrax*) [7–10]. These modifications affect somatic and germinal cells [8] and may play a role during the first steps of domestication [10]. However, the relationships and timeline between genetic and epigenetic modifications remain unclear [6].

All these mechanisms depend greatly on the breeding practices. Indeed, independent trials of domestication may lead potentially to various types of modifications that may have either beneficial (adaptation) or deleterious (maladaptation) effects on various biological functions of the new domesticated populations. The artificial selection, for one or several phenotypes is specific to the domestication process and has consequences that may not be predicted, since morphological, behavioral and physiological traits of animals are intrinsically related [11]. The consequences of modifications of the balance between these traits are not yet properly understood. Indeed, it appears that the artificial selection of specific phenotypes may have deleterious effects on other biological traits because most of resource intakes are dedicated to the selected traits. This imbalance often leads to a decrease of reproduction abilities [5,12], commonly seen in numerous terrestrial and aquatic species. A recent meta-analysis investigating the effect of birth-origin (captive vs. wild) on the reproductive success of animals reared in different anthropogenic environments was performed. For all of the 44 analyzed species, the offspring survival success was decreased in “captive-born” animals compared to their “wild-born” counterparts [13]. It usually involves developmental failures characterized by fertilization issues, embryonic lethalities or the occurrence of deformities. They often ensue from defects of incorporation or synthesis of the eggs’ molecular content. Indeed, the abundance of these molecules, controlling embryogenesis process after fertilization, can directly be affected by modifications of extrinsic or intrinsic factors faced by females during oogenesis [14]. Among them, the maternal mRNA expression profile may thus result from genetic and epigenetic changes in the breeders’ ancestors during the domestication process. It could potentially help to make the link between mechanisms described above and the reproductive success of captive populations in comparison to their wild counterparts.

There are two main ways to study the domestication process [5]. First, longitudinal studies allowing a continuous follow up of a population throughout the domestication process across generations. This method is the most efficient to understand phenotypic and molecular modifications occurring at each step of the domestication process. However, it is long and difficult to perform logistically. The second way, which is commonly used, corresponds to a comparison between wild and domesticated populations. Such method has been previously used and successfully highlighted differences in several fish species, such as steelhead trout (*O*. *mykiss*) [3], Atlantic salmon (*Salmo salar*) [15–17], Atlantic cod (*Gadus morhua*) [18,19], greater amberjack (*Seriola dumerili*) [20] and Eurasian perch (*Perca fluviatilis*) [21,22]. However, numerous pieces of information are often lacking (genetic relatedness between populations, rearing conditions and history of the domestication process) and thus prevent drawing accurate conclusions. Indeed, as previously explained, phenotypic modifications potentially leaded by genetic/epigenetic modifications, depend on rearing practices. Such information is not always tracked properly by farmers. For example, for several domesticated fish stocks, wild breeders are introduced to keep a sufficiently high genetic diversity [23] without keeping track of these introductions leading to incomplete traceability [24]. Today, with the increasing knowledge accuracy and the development of molecular tools, such information becomes important to draw proper conclusions. One way to overcome this issue consists in investigating the genetic differentiation between wild and farmed studied populations. This preliminary step would help understanding differences between populations.

In the context of fish production diversification, numerous efforts are done to domesticate a large number of new species [23]. However, the lack of knowledge on biological and physiological needs of some species may lead to inadequate domestication attempts with deleterious consequences on the biological traits described above. The Eurasian perch *(P*. *fluviatilis)* is a promising species in aquaculture for the production diversification. It is a freshwater fish species widely distributed in Europe and in the Northern part of Asia [25]. It has a niche market with a traditional demand in several European countries [26,27]. The Eurasian perch is a synchronous early spring spawner and its oogenesis induction and progression are mostly controlled by temperature and photoperiod variations [28–33]. Consequently, manipulation of these two factors allowed defining a photothermal program largely used in Eurasian perch farms for out-of season reproduction in recirculating aquaculture systems (RAS) [32]. Despite this successful control of its reproductive cycle allowing out-of-season spawning, the reproduction success remains variable even if the same rearing conditions are applied to the broodstock [34]. It is probably due to the lack of knowledge on potential intrinsic and/or extrinsic modulating factors, including the history and details of the domestication progression experienced by populations.

In the present study, we chose to compare two Eurasian perch populations, reared in the same conditions but with different histories of domestication. We hypothesized that the level of domestication may modulate the accumulation of maternal mRNA in eggs during oogenesis, potentially impacting the embryos early development after fertilization.

## Material and methods

### Origin of fish and broodstock management

Fish were handled in accordance with national and international guidelines for animal welfare protection (*Directive* 2010/63/*EU*. Agreement number: APAFIS#1390–2018031516387833 v2 accepted by the Lorraine Ethic Committee for Animal Experimentation (CELMEA) and the French Ministry of Research). Two populations of three years old Eurasian perch originating from artificial reproductions in November 2011 were used. They correspond to (i) fish at an advanced stage of the domestication process coming from breeders reared in RAS for at least seven generations (F7+ population) and (ii) Eurasian perch originating from breeders collected in the Geneva Lake at the embryonic stage and reared in RAS conditions (F1 population). All animals came from the fish farm “Lucas Perches” (Hampont, France), which provided us the information of the presumable number of generations of the F7+ population and that their ancestors had supposedly been caught in Geneva Lake. Fish were transferred to our indoor facilities after weaning period in February 2012 (mean weight of 3.78 ± 1.07 g). Both populations were reared separately in different tanks but under the same RAS conditions (constant photoperiod (L:D—16:8), 300 lux at the water surface during the lighting period and at 20–21°C) to avoid oogenesis stimulation [32,35] until they reached mean weight of 287 ± 89 g.

**Fig 1:**
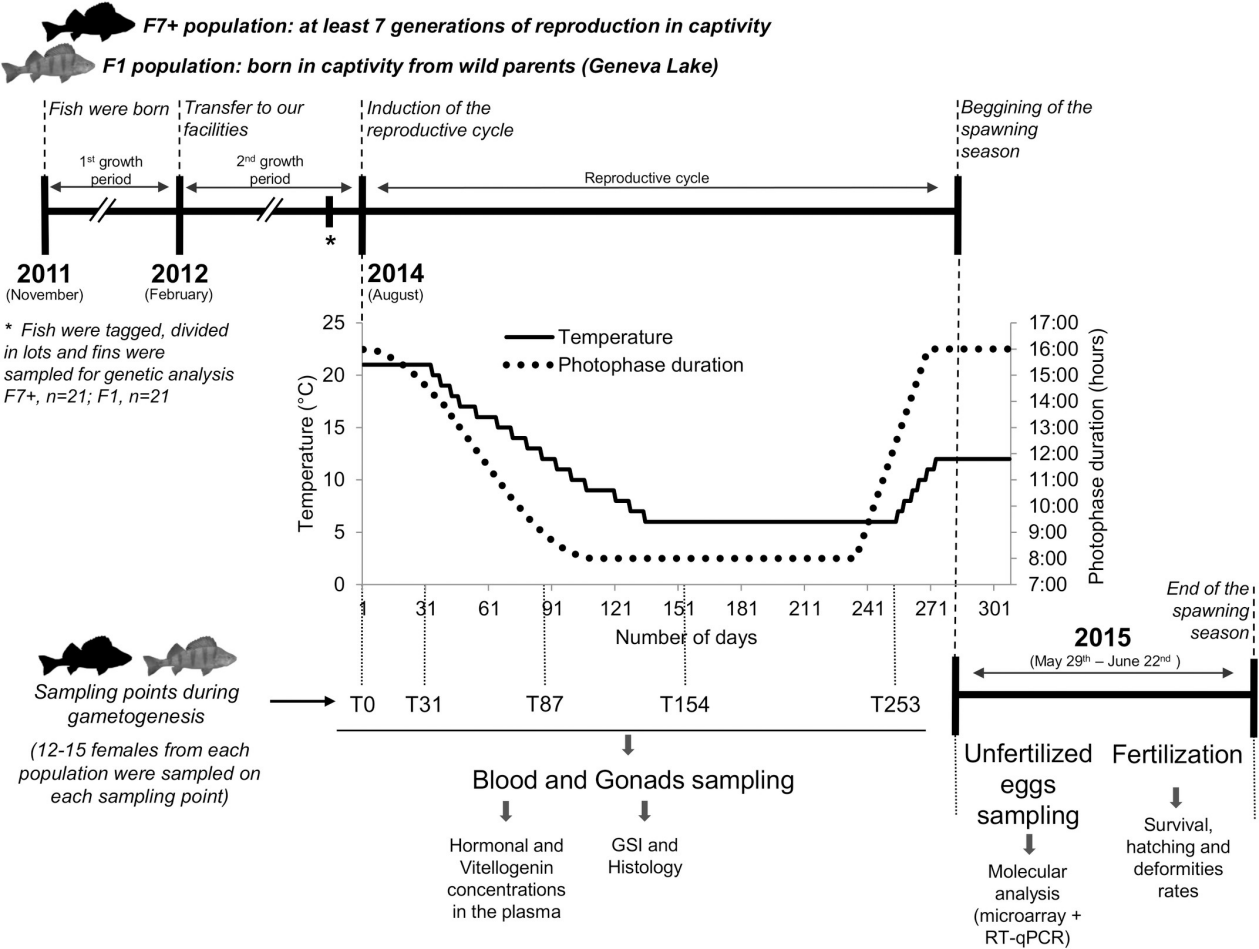
Graphic summary of the experimental design. The photothermal program was used to control each step of the reproductive cycle and spawning of Eurasian perch. Fish sampling was performed at T0, T31, T87, T154 and T253 days after the beginning of the photophase initial decrease (induction of the reproductive cycle). For each sampling point, sexual steroids and Vitellogenin levels were measured in the plasma and a histological follow up of the oogenesis progression was performed. During spawning, each spawn was split into two parts. The first one was not fertilized and frozen for further molecular analyses while the other part was fertilized to assess the developmental progression. All details are given in the method paragraph.

About two months before the experiment began (May 2014), 654 Eurasian perch (313 from the F7+ and 341 from the F1 populations) were transferred to the Aquaculture Experimental Platform (AEP, registration number for animal experimentation C54-547-18) belonging to the URAPA lab and located at the Faculty of Sciences and Technologies of the University of Lorraine (France). They were divided into six independent groups (three per population with an equivalent number of fish in each group). Fish were put into six identical rooms consisting of independent RAS with 3000 liters tanks. Temperature, photoperiod and light intensity were accurately controlled in each room using dedicated software. Environmental conditions were the same during acclimation phase and growing period. In order to induce gonadogenesis, breeders were submitted to a photothermal program allowing effective induction and control of the reproduction cycle [28] from August 18^th^ 2014 to June 22^nd^ 2015 (day 1 to day 309, Fig 1). Water levels of dissolved oxygen (9.53 ± 0.07 mg/L and 9.73 ± 0.12 mg/L), pH (7.55 ± 0.10 and 7.47 ± 0.00), nitrite (0.29 ± 0.14 mg/L and 0.09 ± 0.01 mg/L) and ammonium (0.67 ± 0.22 mg/L and 0.27 ± 0.02 mg/L) were monitored twice a week and kept under the respective thresholds in breeders’ tanks (for F1 and F7+ populations, respectively). At the beginning of the experiment, all animals were individually tagged with P.I.T. tags (Transponder ISO 2 x 12 mm, Biolog-id) to monitor individuals all along the experiment. All fish were fed twice a day to satiation five days a week. In alternation, they were fed three days with commercial pellets (Sturgeon Grower N°5, Le Gouessant) and two days with frozen squids and shrimps (Pomona). On Saturdays and Sundays, they were fed once with commercial pellets to satiety.

### Experimental design, tissue sampling and morphometric measures

In order to follow the oogenesis process, five sampling points of females were performed throughout the reproduction cycle: T0 at the beginning of the experiment allowed us to determine the initial status of breeders, T31, T87 and T154 sampling points allowed us to check the oogenesis along its progression and T253 the final status of the gonads before the spawning season (Fig 1). Four to five females per tank were collected at each sampling point. Firstly, fish were anesthetized by immersion into a Tricaine methanesulfonate solution (120 mg/L; Sigma-Aldrich) for five minutes to collect blood from the caudal vein. Blood was centrifuged at 8000 rpm for 10 minutes in previously heparinized (4.5 mg heparin sodium salt from porcine intestinal mucosa 100KU, Sigma-Aldrich) microtubes. Plasma aliquots were conserved at -80°C for further evaluation of sexual steroids and Vitellogenin concentrations measurements.

After blood sampling, fish were killed using an overdose of Tricaine methanesulfonate (240 mg/L; Sigma-Aldrich) in accordance to European Ethical guidelines (Directive 2010/63/UE). Total weight was measured before collecting the gonads which were weighted to calculate the gonado-somatic index (GSI = gonad weight/total weight*100) and fixed as described below for histological studies.

### Evaluation of steroids and Vitellogenin concentrations in the plasma

The 17-β-estradiol (E2, ng/mL) and testosterone (T, ng/mL) assays were performed on 50 μL of plasma of each sampled female for all sampling points (around 15 females per population/sampling point) using the DIAsource E2-ELISA kit (KAP0621, DIAsource) and the DIAsource Testosterone ELISA kit (KAPD1559, DIAsource), respectively. Samples were diluted from 1/1 to 1/50 for E2 and from 1/1 to 1/10 for T measurements depending on the oogenesis developmental stage. The E2 assay sensitivity was 5±2 pg/mL and the range of use was from 0 to 880 pg/mL with an intra assays CV varying from 4 to 17% and an inter assay CV of about 27%. Concerning T, the sensitivity was 0.083 ng/mL and the range of use was from 0 to 16 ng/mL. The intra assays CV varied from 7 to 19% and the inter assay CV was about 16%.

The Vitellogenin plasmatic concentration was indirectly estimated in 80 μl of plasma by measuring concentrations of the alkali-labile phosphate level as described in [36].

### Gonads histology

Female gonads from all sampling points were fixed in Bouin’s solution for one week before being washed in 70% alcohol. Samples were then dehydrated in ascending series of ethanol (70–100%) before being embedded in paraffin with an orientation allowing transversal cuts. Five micrometer sections were performed with a Leitz Wetzlar microtome and collected on glass slides. Masson’s trichrome staining was done according to a protocol adapted from [37] as follows: Hematoxylin solution modified according to Gill III (Merck) was used from five to ten minutes; Phloxine (VWR) was diluted in water at 0.5% and used for five minutes; Light Green (Sigma) was diluted in water at 0.5% and used from three to five minutes. Stained sections were examined, photographed and analyzed using a light upright optical microscope (Nikon Eclipse Ni-U) associated with a DS-Fi1 digital camera and the software NIS BR (Nikon France, Champigny-sur-Marne, France) at low magnification (x2 and x4).

Oocytes stages were determined according to [38] and classified into six classes: primary growth (PG), early cortical alveoli stage (ECA), late cortical alveoli stage (LCA), early vitellogenesis (EV), late vitellogenesis (LV) and atresia (A). Oogonia (O) stages were also identified.

The gonadic maturation state was determined by counting all oocytes of each class on one complete and representative transversal stained sections of the ovary for T0-T154 and three representative transversal sections for T253 because the gonads were then too large to be laid on one slide.

### Gamete collection and fertilization

Before the spawning season, all females were caught to take some oocytes using a catheter and determine their oocytes maturation according to [39]. On May 13^rd^ and 15^th^ 2015, females from all tanks were examined and were allocated to separate tanks for the spawning season, according to their oocyte maturation stage and regardless of their original population. Thus, one tank contained the females having oocytes at stages I and II, another tank contained the females at stages III and IV (which were all coming from the F1 population), and another tank contained females that could not be staged reliably. Males from F7+ and F1 populations were kept apart in two tanks depending on their origin. When one female spawned, all females of this tank were monitored daily to identify ovulation and collect the eggs by stripping them. This procedure was always performed between 4am and 5am and each spawn was treated individually. The first spawn observed in each tank was not considered for the experiment because once in the water, the eggs are activated and their ability to be fertilized decrease rapidly. The spawning season took place from May 29^th^ to June 22^nd^ 2015 and each female stripped was identified as to its original population by its P.I.T. tag. Eggs were fertilized as described in [40] with sperm from three males (total volume of 100 μl sperm/g dry eggs). Eggs stripped in the same day were fertilized using the same pool of sperm and at the end of the spawning season no day effect was seen in the reproductive performance. All males used to perform fertilization came from the F1 population, that presented a higher quality (motility estimated under a light upright optical microscope (Nikon Eclipse Ni-U)). The other part of each spawn (18 individual samples of about 200mg) was immediately frozen in liquid nitrogen and kept at -80°C for subsequent transcriptomic analysis.

### Study of reproductive performance

In total, 32 spawn, corresponding to 32 females (13 from F7+ population and 19 from the F1), were treated as described above. A previous study performed in our team showed that cell cleavage timetable can be highly variable between embryos even within the same spawn [41]. Thus, we choose to perform our first evaluation of the embryonic survival at 24 hours post fertilization (hpf) because it was the earliest stage to establish a relevant estimation of survival. We were thus not able to make a distinction between non-fertilized eggs and dead embryos. Eurasian perch eggs are surrounded by a jelly coat that protects embryos from the outside environment [29]. This jelly coat prevents to sort the eggs depending on their survival at each stage as it is currently performed with other fish species for which eggs are not attached. Thus, we performed the following protocol to evaluate the survival in the same ribbon samples at several timepoints.

Just after fertilization, three ribbon fragments (samples) of around 500 eggs were randomly cut from each spawn/ribbon and incubated to determine the percentage of embryos alive at different timepoints. We considered that the borders of each fragment should be avoided since embryonic development in this region could be impacted by the cutting. At each specific timepoint, around 100 embryos were counted and, among them, the number of alive embryos (those reaching the proper developmental stage) was counted to evaluate the percentage of alive embryos. These evaluations were performed in the middle of each fragment at 24, 48, 72 and 120 hpf to avoid potential border effects described above. The percentage of embryos alive was estimated using the following formula: (number of alive embryos at one stage/total number of embryos studied at that stage) x 100. In addition, three other fertilized samples of around 100 eggs/sample were kept apart, without manipulation, until the hatching period. They were used to determine the percentage of embryos hatching using the following formula: (number of hatched embryos/total number of embryos in the fragment) x 100 and deformities rates according to [42]. The global deformities rate (Dr) and specific deformities rates allowed studying defects in the axis (Ad), yolk (Yd), cardiac (Cd), mouth (Md), eyes (Ed) and others (Od) as described in [42].

### RNA extraction

Total RNA was extracted from unfertilized frozen eggs from the 32 spawn (mean weight of 100 mg, 10–15 eggs) using TRIzol reagent (Life Technologies) at a ratio of 100mg per mL of reagent and following the manufacturer's instructions with some modifications. Indeed, a milling step was added during the homogenization step to get rid more easily of the gelatinous envelope and chorion surrounding eggs. To do so, a bullet blender (Next Advance) and zirconium oxide beads 1.0 mm were used. In addition, a supplementary centrifugation (4°C, 30min. 13000 rpm) was performed before the addition of the chloroform to remove the lipid content in eggs. A NanoDrop ND-1000 Spectrophotometer (NanoDrop Technologies) was used to evaluate the quantity of total RNA and an Agilent 2100 Bioanalyzer (Agilent Technologies) was used to evaluate the integrity of the RNA extracted. Samples exhibited an integrity score higher than 7 and were used for the microarray analysis.

### Microarray analysis

The Eurasian perch array (SurePrint G3 Custom Gene Expression Microarray, 8x60K - Agilent Technologies) contains 48,986 non-redundant probes previously identified and available from the PhyloFish Database [43]. The “One-Color Microarray-Based Gene Expression Analysis (Low Input Quick Amp Labeling) Protocol” was followed for samples preparation, hybridization, washing and scanning of slides and data extractions. Briefly, 150 ng of total RNA were used for the amplification/Cy3-labeling step. After this step, samples were purified (RNeasy mini kit, Qiagen) and quantified using a NanoDrop ND-1000 Spectrophotometer (NanoDrop Technologies). Samples exhibiting a yield higher than 1.03 μg of cRNA and a specific activity higher than 10.30 pmol of Cy3/μg of cRNA were fragmented and used to hybridize arrays (one sample failed the labeling step and was excluded from the experiment). Samples (600 ng of Cy3-cRNA) were randomly distributed onto four slides. After 17h of hybridization at 65°C, slides were washed, dried and scanned with an Agilent Technologies Scanner (G2505C). Scanned images were extracted with Agilent Feature Extraction software.

Data extracted from scanned images were normalized and log(2) transformed for statistical analyses (all data are available in the Gene Expression Omnibus database under the accession code GSE119802). Using the GeneSpring software, an unpaired t-test followed by a Benjamini-Hochberg correction was used to identify genes differentially expressed (DEG) between the two populations (p<0.05). Then, a hierarchical clustering analysis (unsupervised average linkage) was performed using Cluster 3.0 software (version1.52). Clusters were visualized using TreeView software (version 1.1.6r4).

### Gene ontology analyses

In order to give an overview of gene ontology (GO) terms represented among differentially expressed genes between F1 and F7+ populations, they were functionally annotated and classified using Blast2GOv4.1.9 software [44]. Default parameters were used for blastx and GO annotations.

In the following, we performed a GO overrepresentation analysis in which we compared the list of DEG to a reference list corresponding to all expressed genes in the microarray. To retrieve this reference list, we first filtered all genes present in the array. Genes were, considered as expressed when they presented a signal above background in at least 75% of the samples and in at least one of the populations. In a second time, as the Eurasian perch genome is not yet available in any databases allowing performing GO analyses, we chose to retrieve human orthologs identifiers for the reference and DEG lists. It allowed us to perform the analysis. In order to find these identifiers, we aligned probes designed for the array corresponding to each sequence in the lists against the Eurasian perch transcriptome extracted from the genome, provided by [45], with minimap2 (version 2.7 with -m 20 parameters). Because the gene prediction file had small UTRs, we extended the prediction on both transcript sides by 2kb. If a probe had a unique alignment, it was assigned to the corresponding transcript. If the probe had two corresponding transcripts located one after the other on the genome, we assigned it to the transcript having the match closest to its center. Probes with no match or over two matches were not assigned to a transcript. We then retrieved the human orthologs identifiers from the annotation file provided by [45]. The genes still missing identifiers in the DEG list were manually annotated using UniProt accessions. In total, among the 358 DEG, 265 human orthologs identifiers were found. The GO overrepresentation analysis was performed using Panther14.0 [46]. Parameters were set at default, meaning that a Fisher’s exact test and a Benjamini-Hochberg correction were applied. We asked for GO-Slim Biological Processes (BP) and Pathways and only corrected p-values <0.05 were considered as significant.

### Real-time PCR analysis

Genes presenting a log(2) fold change (log(2)FC) > 4 in microarray were additionally studied by real-time qPCR in all samples previously used for the microarray. After RNA extraction, a DNase treatment (DNase I, RNase free—Thermo Scientific) was applied to 5 μg of all samples (n = 32) following the manufacturer’s protocol. The reverse transcription was performed in a final volume of 20 μl using a M-MLV Reverse Transcriptase (Sigma-Aldrich), 1 μg of RNA and random nonamers (2.5 μM—Sigma-Aldrich) and following the manufacturer’s protocol. Reverse transcript products were diluted 1:27 and 5 μl were used for the real-time PCR, using PerfeCTa SYBR Green SuperMix (Quanta Bioscience) and 5 pmol of each primer. Primers were designed using Primer3Plus or Primer design Tool-NCBI software (S1 Table). The real-time qPCR was performed using a Step One Plus system (Applied Biosystems, Foster City, USA). The PCR program consists of a first step at 95°C for two minutes followed by 40 cycles consisting of a denaturation step at 94°C for 15s, an annealing step at 50–58°C depending on the primers pairs for 15s and an elongation step at 72°C for 30s. The amplification was followed by a melting curve stage, according to manufacturer’s parameters in order to check the primers specificity. The abundance of the target cDNA in each sample was calculated using a serial dilution of a pool of all cDNA samples using the StepOne Software (Applied Biosystems, version 2.1). This dilution curve was used to certify the reaction efficiency (80–120%). All samples were analyzed in duplicate and the geometric mean of the expression level data of the *adenosine kinase-like*, *RNA18s*, *TATA-box binding* and *ELAV-like protein 1-like* (S1 Table) was used as reference to normalize the data obtained. These genes were found as stable in microarray and preliminary real-time qPCR analyses in all samples. The values obtained after normalization were analyzed using a Mann-Whitney test (RStudio software version 1.0.143) to compare differences of gene expression between populations (p<0.05).

### Genetic variability between populations

Genomic DNA was extracted from 42 fin samples representing the two populations (21 F1 and 21 F7+), using the universal salt-extraction method, according to [47]. Purity and quantity of genomic DNA were assessed using a NanoDrop ND-1000 Spectrophotometer (NanoDrop Technologies). Eight microsatellites previously used on *P*. *fluviatilis* [48] were selected: *PflaL1*, *PflaL2*, *PflaL4*, *PflaL6* [49], *SviL7* [50], *Svi17* [51], *YP60* and *YP111* [52]. Two multiplex amplifications were done using fluorescently labeled primers. The first multiplex (A) contained *PflaL2* (FAM), *PflaL4* (PET), *SviL7* (VIC), *Svi17* (FAM) and *YP111* (PET). The second one (B) contained *YP60* (FAM), *PflaL1* (VIC) and *PflaL6* (FAM). Polymerase chain reaction was carried out using the Multiplex TEMPase 2X MasterMix (VWR), 10 pmol of fluorescent primer mix, genomic DNA and water for a final volume of 30 μl. PCR conditions for multiplex A were: 95°C for five minutes, 28 cycles at 95°C for 30 seconds, 55°C for 90 seconds and 72°C for 30 seconds, and a final extension of 45 seconds at 60°C. For multiplex B, PCR conditions were: 95°C for five minutes, six cycles at 95°C for 30s, 48°C for 90s and 72°C for 30s, 22 cycles at 95°C for 30s, 50°C for 90s and 72°C for 30s, and a final extension at 60°C for 45s. PCR products were diluted (1:151) with deionized water and added Hi-Di^™^ Formamide (Applied Biosystems) and GeneScan 600 LIZ Size Standard (Applied Biosystem). The fragment analysis was performed on a 3500 Genetic Analyzer (Applied Biosystems HITACHI) and alleles were scored with Geneious 11.0.2 [53].

Genetic diversity was estimated through calculation of observed (Ho) and expected (He) heterozygosities in GENETIX [54]. Population differentiation was assessed by estimating the “global” F_ST_ statistic on populations through an analysis of molecular variance (AMOVA when considering only one group of populations) performed in Arlequin with 10000 permutations. Divergence between populations was estimated with a F_ST_-pairwise test (10000 permutations).

### Statistical analysis

Differences in GSI, sexual steroids and Vitellogenin concentrations, oocytes stages abundance on histological cut and specific deformities were estimated using a non-parametric Wilcoxon-Mann-Whitney. Normality and homogeneity of variance were tested using respectively Shapiro-Wilk and Levene’s test.

In order to investigate possible differences of embryonic development between populations, percentage of embryos alive at 24, 48, 72 and 120 hpf and at hatching were compared between populations using a one-way repeated-measures ANOVA. The statistical model included as fixed effect the percentage of alive embryos for F1 and F7+ populations. It has been controlled by the introduction of the females as covariate. This model was chosen after comparison with a second one without a covariate. They were ranked according to their Akaike information criterion and the one having the lowest criterion was chosen [55]. A TukeyHSD was performed to identify differences between populations at different times individually.

For all tests, a p-value ≤ 0.05 was considered statistically significant. All values given are represented as means ± standard error of mean (SEM). All tests were performed using R (v.1.1.423) [56]. The package *stats* was used to perform Shapiro-Wilk, Wilcoxon-Mann-Whitney, ANOVA and TukeyHSD tests. While *car* was employed for Levene’s test.

## Results

Two Eurasian perch populations (F1 and F7+) were used in the present study. Analysis of their genetic differentiation using microsatellites revealed a F_ST_ of 0.1055 (p < 0.001). In addition, the F7+ population presented a higher observed heterozygosity (mean = 0.440) compared to the F1 (mean = 0.348), indicating a larger genetic diversity of the F7+ population.

### Follow up of the gonadogenesis progression reveals few differences between populations

Five sampling points of females were performed as shown in Fig 1. The GSI of both populations increased progressively all along gonadogenesis to reach close to 13% one month before the spawning season (S1A Fig). The only difference between populations can be seen at T31 with a higher GSI (p = 0.02) for the F7+ females (0.91 ± 0.05%) compared to the F1 ones (0.69 ± 0.06%). A follow up of the hormonal status of females did not present any significant difference of the testosterone level between populations. At T253 a higher level was recorded for the F1 (50.35 ± 5.89 ng/mL) compared to the F7+ (35.81 ± 6.49 ng/mL, p = 0.06 –S1B Fig). Similar data were obtained for the 17-β-œstradiol (E2), except for the T253 for which the hormonal level was higher (p = 0.03) for F7+ (8.71 ± 0.34 ng/mL) than for F1 (7.39 ± 0.44 ng/mL), no other statistical difference was found (S1C Fig). The follow up of the Vitellogenin level in the blood did not present any significant difference between F7+ and F1 populations (S1D Fig).

**Fig 2:**
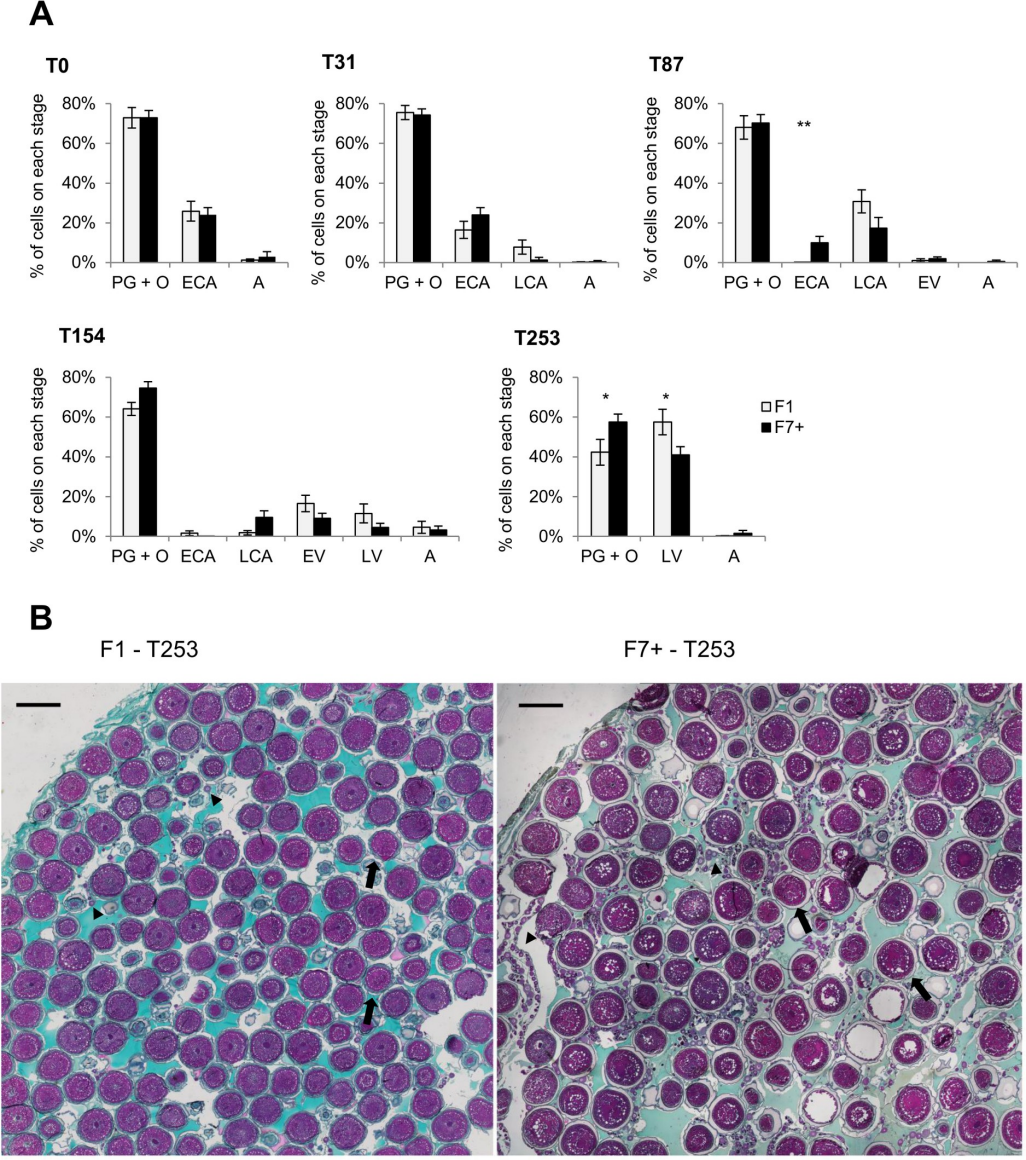
Histological follow up of the oogenesis progression. (A) Percentage of each cellular stage in gonads belonging to F7+ and F1 populations during oogenesis period. Stages are represented as: primary growth and oogonia—PG+O, early cortical alveoli—ECA, late cortical alveoli stage—LCA, early vitellogenesis—EV, late vitellogenesis—LV and atresia—A. Differences between the two populations were tested using non-parametric Wilcoxon-Mann-Whitney test (p<0.05). Significance levels are represented as follows: *, p = 0.05–0.01; **, p = 0.01–0.001; ***, p = 0.001–0.0001; and ****, p < 0.0001. (B) Histological section of gonads representative of F7+ and F1 ovaries at T253. Arrows points to LV stages and arrowheads indicate the PG+O stages. Scale bars represent 1000 μm.

These data suggest that the oogenesis progression for both populations was similar. However, the histological study of gonads revealed that F1 females presented higher percentage of late vitellogenesis oocytes (57 ± 6%) at the end of the oogenesis in comparison to the F7+ ones (41 ± 4%; T253, Fig 2A and 2B), suggesting that oogenesis was slightly more advanced in the F1 than in the F7+.

### The embryonic survival is higher in F1 than in F7+ spawn

**Fig 3:**
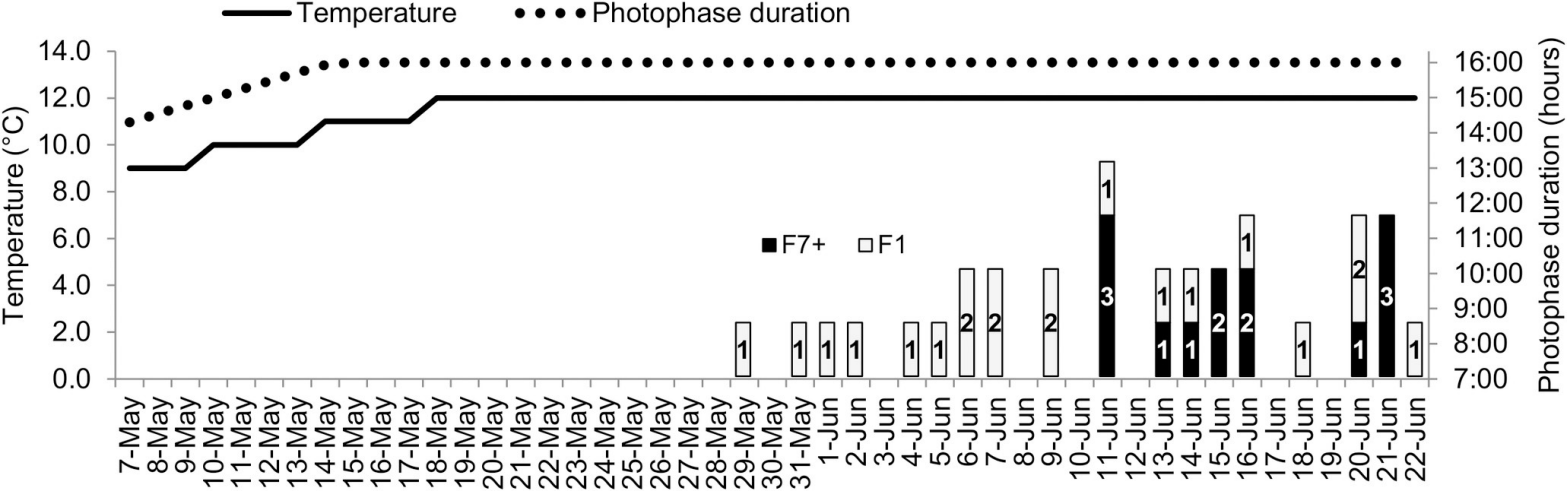
Timetable of the spawn obtained for both populations during the spawning season in relation to temperature and photophase increase at the end of the photothermal program. Bars with numbers correspond to the number of spawn obtained each day for F1 and F7+ females.

Following the observation of a slightly faster oocytes development in F1 population, the first spawning was more precocious for F1 than for F7+ fish. F1 females, coming from all three original replicate tanks, spawned between thirteen and seven days earlier than F7+ first spawner (Fig 3).

The main effect for population yielded an F ratio of F(1,30) = 4.266, p = 0.0476, indicating a significance difference on the number of embryos alive depending on the population.

**Fig 4:**
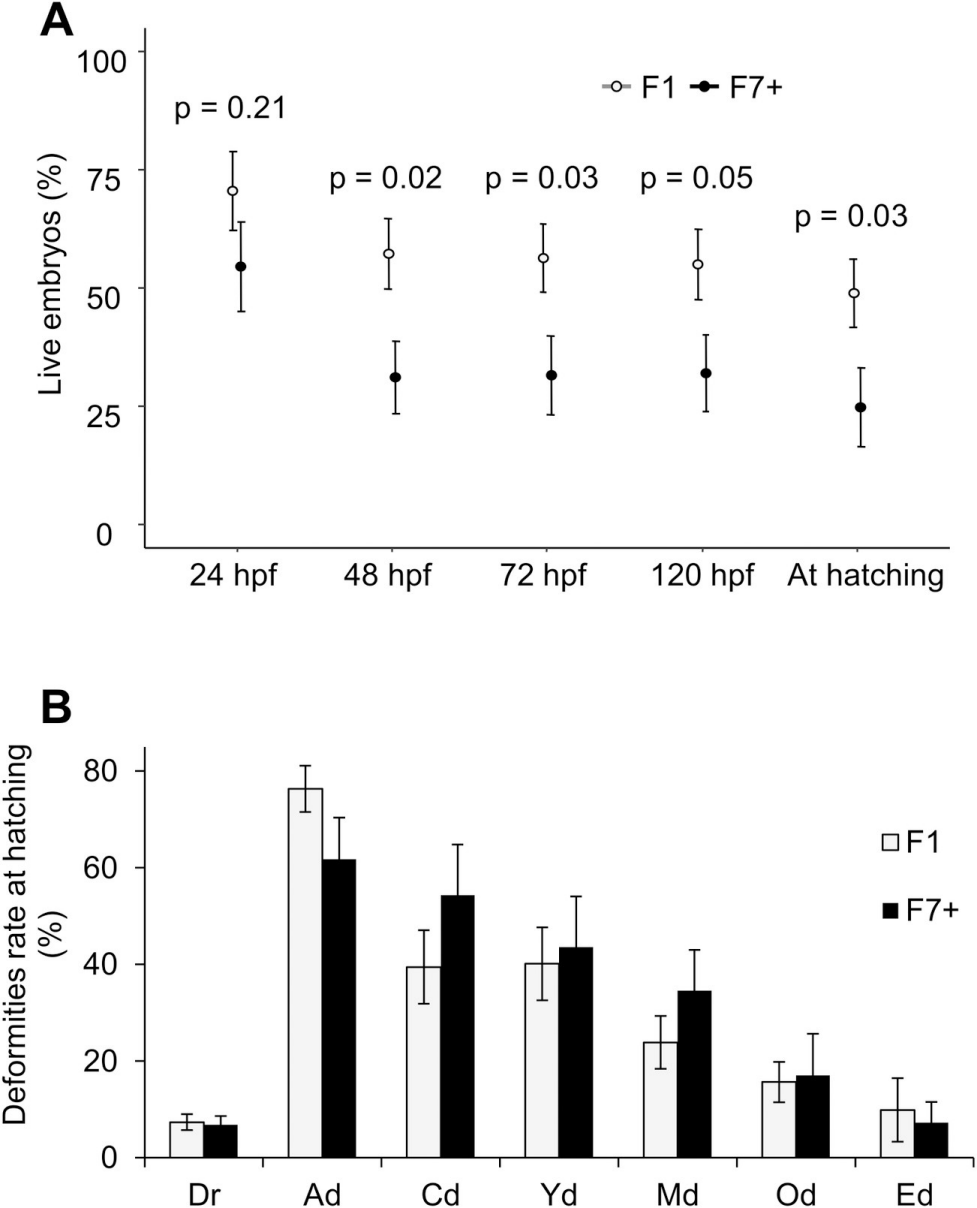
Evaluation of the embryonic developmental success in F1 and F7+ populations. (A) Percentage of embryos surviving at 24, 48, 72 and 120 hours post-fertilization (hpf) and at hatching in F1 (light gray dots) and F7+ (black dots) populations estimated based on the total number of studied embryos (about 100 embryos). In (A), dots represent population means ± SEM at each observed time (n = 19 and 13 for F1 and F7+, respectively). P-values presented represent comparisons between populations at each time of observation obtained using a TukeyHSD preceded by a significant one-way ANOVA in repeated measures (p ≤ 0.05 were considered as significant). (B) Total deformities rates (Dr) and specific rates in various tissues at hatching in F1 and F7+ populations (Ad—Axis, Cd—Cardiac, Yd—Yolk, Md—Mouth, Od–Other, Ed—Eyes). No significant difference has been identified between populations using non-parametric Wilcoxon-Mann-Whitney test (p<0.05; n = 23).

Since an overall population effect on the percentage of embryos alive was observed, TukeyHSD tests were performed between populations and revealed significant differences between them at 48 (p = 0.02), 72 (p = 0.03) and 120 hpf (p = 0.05) and at hatching (p = 0.03). Consequently, embryonic survivorship was significantly higher in F1 than in F7+ population from 48 hpf (Fig 4A and S2 Table). Interestingly, more heterogeneity of survivorship is seen in F7+ population at all timepoints (coefficient of variation (CV) = 62%, 88%, 95%, 90% and 121% at 24, 48, 72 and 120 hpf and at hatching, respectively) in comparison to F1 in the same stages (CV = 51%, 56%, 56%, 59% and 64% at 24, 48, 72 and 120 hpf and at hatching, respectively). The overall occurrence of deformities and that of specific deformities were comparable between populations and did not present any statistical difference (Fig 4B and S3 Table).

### Eggs transcriptomic analysis

**Fig 5:**
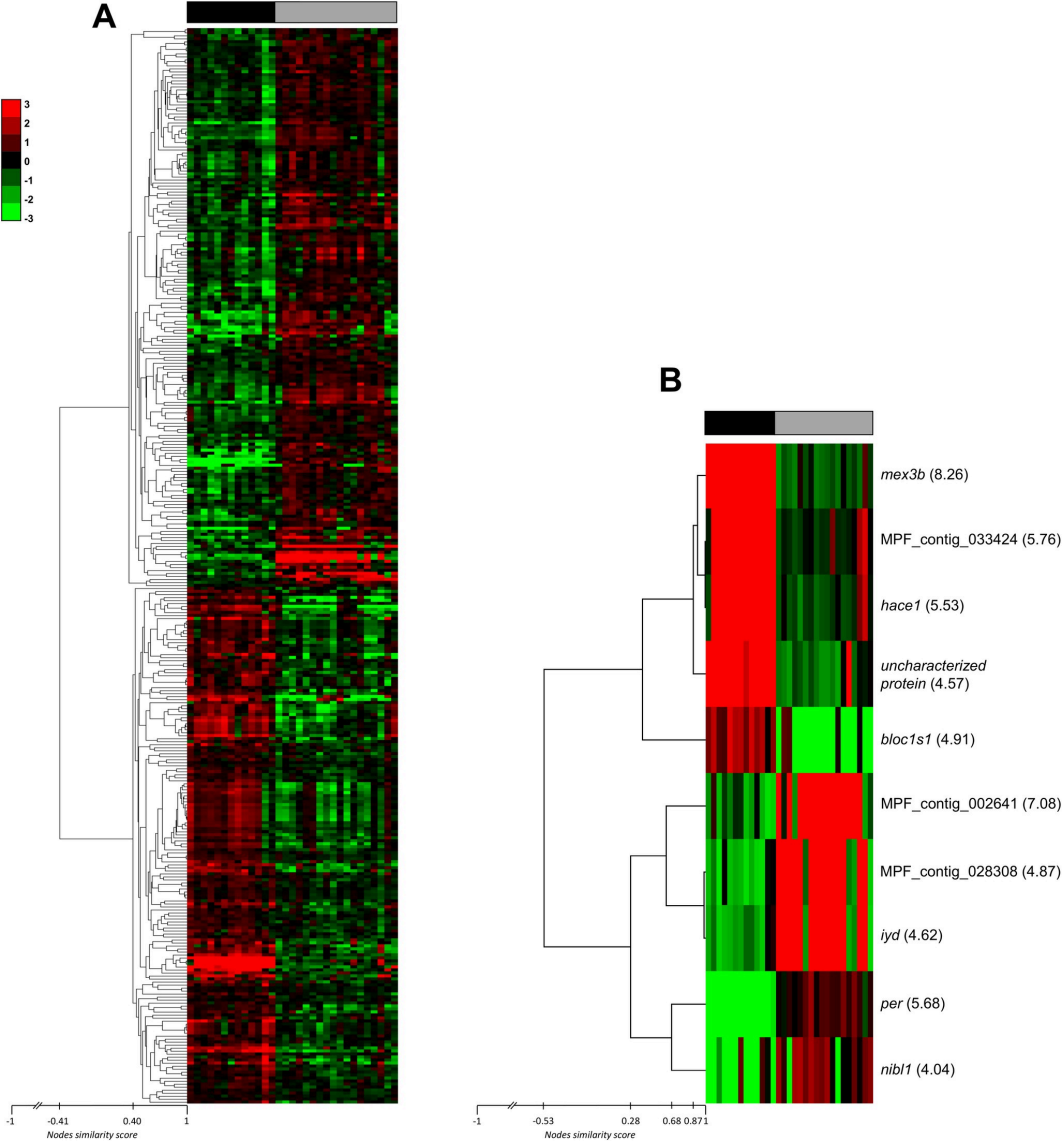
Heatmaps showing differentially expressed genes between F1 and F7+ populations. (A) Unsupervised hierarchical cluster analysis of 358 differentially expressed genes (p < 0.05) between populations. The dendrogram on the left represents gene correlation distances between the genes. (B) Unsupervised hierarchical cluster analysis of the 10 most differentially expressed genes (p < 0.05) with a log(2)FC >4, between populations. Gene abbreviated name and log(2)FC are shown between parentheses on the right. In both parts of the figure, red color indicates over-expression, and green color indicates under-expression while black is used for median expression. Top bar indicates the origin of the samples: black for F7+ samples and gray for F1 ones. Node similarity score bars represents the similarity between tree branches. It ranges from 1 (identical) to -1 (opposites), while 0 means they are completely uncorrelated.

A large scale analysis was performed on 31 spawn to compare the maternal transcriptomic profiles of non fertilized eggs. The statistical analysis revealed 358 differentially expressed genes (DEG) between populations (S4 Table). An unsupervised average linkage clustering analysis was performed using the expression data of these 358 DEG. It allowed splitting apart both populations revealing that 172 genes were over-expressed in the F7+ population and 186 genes were over-expressed in the F1 population (Fig 5A and S5 Table).

A functional annotation of these 172 and 186 over-expressed genes allowed mapping 86 genes for each population, among which 73 and 76 genes containing specific GO annotations were identified for F7+ and F1 populations, respectively (S6 and S7 Tables).

Using Panther software, no Pathways was enriched and the term “immune system” was overrepresented (FDR p-value = 0.00251; S8 Table). Four of the genes represented belong to the butyrophilin or the butyrophilin-like family but two of them were up-regulated in the F1 population while the others were down-regulated in this population. For three of these genes, the log(2)FC was >2 and were thus among the 16% most differentially expressed genes between the two populations.

Indeed, among the 358 DEG only 60 presented a log(2)FC > 2 between the two populations. Fifty DEG showed a log(2)FC between 2 and 3-fold (S4 Table) and 10 genes had a log(2)FC higher than 4-fold (5 over-expressed in the F7+ population and 5 in the F1; Fig 5B). We choose to check the expression level of the genes having a log(2)FC > 4 by real-time qPCR. Among them, three sequences could not be identified because the probes actually recognized contigs grouping numerous unidentified genes in the Eurasian perch transcriptomic database (Fig 5B). Among the remaining genes, four were more expressed in the F7+ population: *mex3b* (log(2)FC = 8.26), *bloc1s1* (log(2)FC = 4.91), an *uncharacterized protein* (log(2)FC = 4.57) and *hace1* (log(2)FC = 5.53). In the same way, *per2* (log(2)FC = 5.68), *nibl1* (log(2)FC = 4.04) and *iyd* (log(2)FC = 4.62) were more abundant in the F1 population.

**Fig 6:**
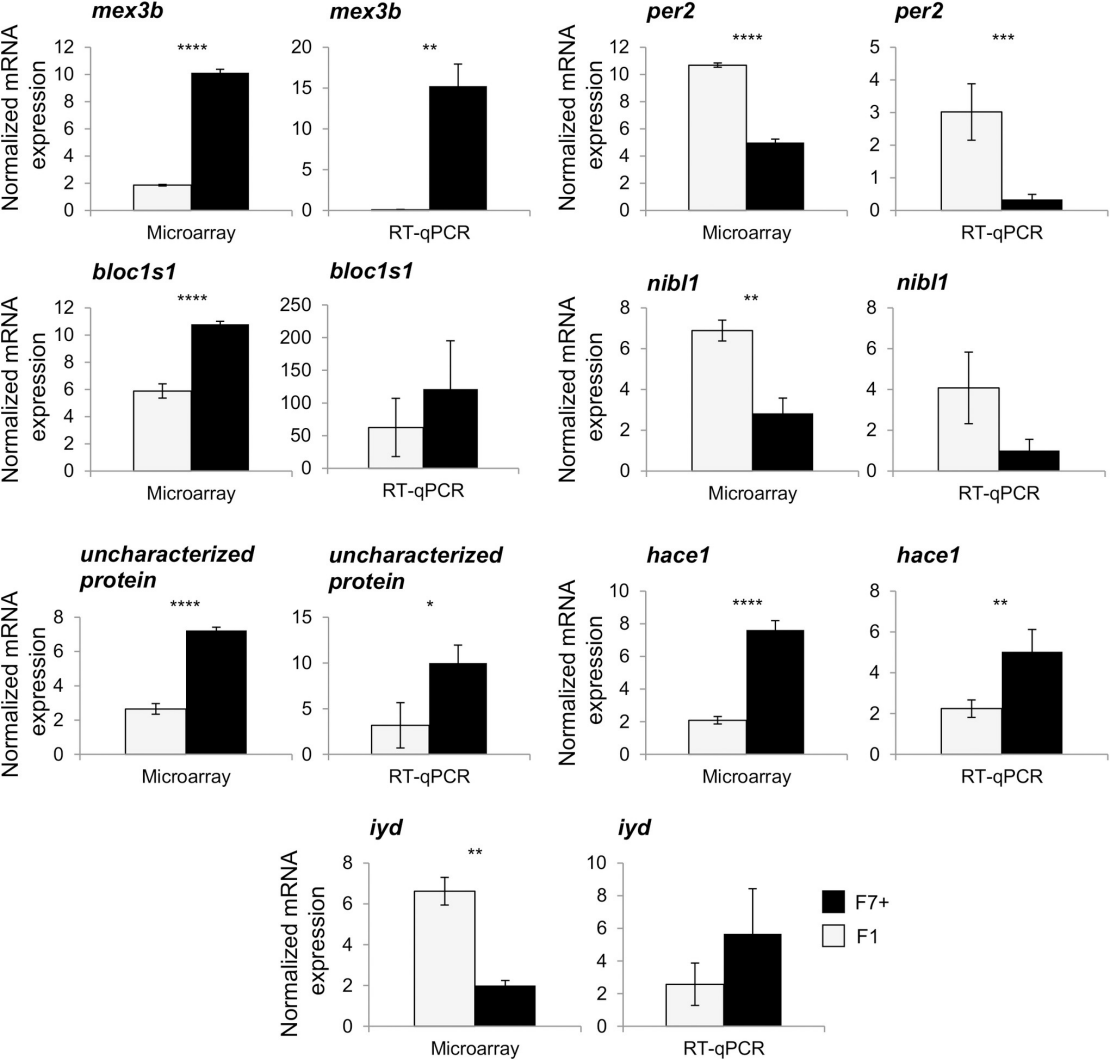
Compared analysis of seven genes among the most differentially expressed (log(2)FC >4) between microarray and RT-qPCR experiments. The same egg samples were used to perform both techniques for F7+ (n = 13) and F1 (n = 18 for microarray and n = 19 for RT-qPCR) populations. An unpaired t-test followed by a multiple testing correction Benjamini-Hochberg was applied on microarray results while a Mann-Whitney-Wilcoxon test was used on RT-qPCR results. Bars correspond to the means ± standard error. Significance levels are represented as follows: *, p = 0.05–0.01; **, p = 0.01–0.001; ***, p = 0.001–0.0001; and ****, p < 0.0001.

Expression level differences were confirmed for *mex3b* (log(2)FC = 7.26 for the RT-qPCR), the *uncharacterized protein* (log(2)FC = 1.65 for the RT-qPCR), *hace1* (log(2)FC = 1.65 for the RT-qPCR) and *per2* (log(2)FC = 3.13 for the RT-qPCR; Fig 6). However, for *bloc1s1* and *nibl1* the expression levels between both populations were not significantly different by RT-qPCR even if they followed the same profile than in the microarray (Fig 6). In addition, concerning *iyd* not only no significantly different expression was observed, but also the profile observed by RT-qPCR was in favor to a higher expression in the F7+ population, which is in contradiction with the microarray data.

## Discussion

The Differentiation Index (F_ST_) illustrates a genetic divergence between populations ranging from 0 (gene flow between populations) to 1 (isolated populations without any gene flow). In the literature, a lower F_ST_ (0.002, p < 0.0023) was found between various samples of Eurasian perch all around the Geneva Lake [48] and authors considered that there was only one population in the lake. The same authors showed a minimum F_ST_ of 0.45 (p < 0.0001) between a wild and nine farmed populations of Eurasian perch that were all supposed to originate from the same geographic location [24]. They were considered as distinct populations. In comparison, our F_ST_ (0.1055, p < 0.0001) is in between the above mentioned examples. The interpretation of significant differences when using highly variable loci, such as microsatellites, has to be very carefully analyzed since its biological meaning can often be weak [57]. In the present study, we selected the most variable microsatellites according to literature on Eurasian perch microsatellite differentiation. In addition, the higher heterozygosity index for the F7+ (0.440) compared to the F1 (0.348) shows a higher heterogeneity in the F7+ population compared to the F1. Our F7+ population came from a partner fish farmer but first originated from the transfer from another fish farm. The initial stock was supposedly from Geneva lake but it was difficult to obtain a reliable traceability. Consequently, it is possible that some individuals from different geographic locations may have been introduced in the F7+ stock, which is a common practice in aquaculture [24]. Alternatively, the random independent sampling process to create each population stock (F1 and F7+) could have led to different degrees of genetic drift in each stock. In our conditions, none of these hypotheses can be ruled out.

### Control of the reproductive cycle

The oogenesis progression was similar between populations even if females from the F1 population seemed to respond faster to the photothermal stimulation and kept a slight non-significant advance all along the process. This advance became significant one month before the spawning with a higher proportion of late vitellogenesis oocytes and may contribute to explain the early onset of the spawning season of F1 females (thirteen to seven days before the F7+ ones). For technical reasons it has not been possible to determine the end of the spawning season of F7+ fish. The short spawning season of F7+ population in comparison to F1 is thus not relevant. It is worthy to note that at the end of the experimental period all remaining females that did not spawn presented developed gonads, once they were all slaughtered at the end of the experiment.

In this study, sexual steroids and Vitellogenin levels were either slightly or not different during the reproduction cycle between populations. The same pattern has been observed for the GSI. Differences found may either be mediated by yet unknown mechanisms or very subtle variations of hormone levels controlling the oogenesis progression. From the current knowledge, it remains difficult to precisely point which of these parameters imposed these modulations between the two populations.

Concerning the developmental success, results showed that F1 population presented an embryonic survival higher than the F7+ one with differences ranging from 16 to 26% of survival depending on the timepoint. This difference is the lowest at 24 hpf (16%), leading to a non-significant result at this timepoint. In any case, differences between populations may be due to egg quality issues that led either to fertilization impairments or higher mortality occurrence. These results enable us to conclude that the overall egg quality was higher in F1 than in F7+ population. Egg quality relies greatly upon its intrinsic content and some molecules may have very early and essential effect during embryogenesis, including pronuclear congregation and mitotic spindle assembly [58]. In addition, only few exchanges occur between the developing embryos and the environment [34] reinforcing the importance of the egg content during early embryogenesis. Moreover, the F7+ population shows more heterogeneous survivorship. It is potentially related to individuals’ history in the tank during the oogenesis (e.g. behavioral difference). In any case this difference of egg quality may be explained by the resource allocation theory [12]. Even if domestication of Eurasian perch begun 20 years ago, numerous questions remain to properly achieve their breeding in recirculating aquaculture systems. Since no selective program has begun yet for this species [23], the artificial selection driven by farmers remains empirical. In addition, rearing conditions are not fully optimized and fish farmers continue to make changes in their practices. Consequently, an imbalance between biological functions may occur in this species because resource intakes are allocated toward certain traits at the expense of other functions, such as reproduction. No zootechnical practices may be undertaken to compensate the lack of intake that may occur at each generation. This data is in accordance with numerous studies showing a decrease of the reproductive success in domesticated populations. Indeed, lower fertilization and hatching rates in farmed Atlantic cod (*G*. *morhua*) [18,19], lower survival rate at the eyed stage in farmed Atlantic salmon (*S*. *salar*) [59], lower hatching rate in cultured common sole (*Solea solea*) [60] and oogenesis impairments in captive-reared greater amberjack (*S*. *dumerili*) [20] were described in comparison with their respective wild counterparts. Therefore, this problem is widely observed in fish species and further studies are needed to test the hypotheses proposed in the resource allocation theory during fish domestication. If these hypotheses are confirmed, it means that a more accurate knowledge of metabolic needs for each biological function at each step of the life cycle is required before starting any selection program. Farmers should then take this information into account in order to implement a compensation program when the resource allocation is imbalanced. In this manner, all biological functions may beneficiate from an increase of the global resource intake. In addition, the choice of selected biological traits in addition to the follow up of the fitness of individuals should be assessed properly in order to detect early any deviation.

Concerning the Eurasian perch (*P*. *fluviatilis*), other studies showed that wild populations had higher reproductive performance than captive ones but with even more drastic differences than in the present work [21,22]. Differences between our study and these ones may be due to accumulation of the domestication effect on oogenesis and spermatogenesis, since in those studies developmental performance of embryos was evaluated from pure crosses from each population. In the present study, our goal was to investigate exclusively the effect of the domestication on females’ performance. Consequently, we chose to reduce the potential effect of sperm quality and fertilized all spawn with sperm from F1 males. Another explanation could be that rearing conditions using in other studies were different (tank size and colour, first spawn or not…) from ours and influenced differently wild populations in the different studies.

Variations of gene expression profiles between wild and “domesticated” populations have been demonstrated in whole larvae and embryos of Atlantic salmon (*S*. *salar*) [15,61], fertilized eggs of Atlantic cod (*G*. *morhua*) [18] and whole larvae of steelhead trout (*O*. *mykiss*) [3] but no study investigated the maternal mRNA profile. These observations strengthen the necessity to better understand the contribution of maternal mRNA to the embryonic early development in our conditions.

### Eggs transcriptome

Our working hypothesis was that the domestication process may impact the maternal mRNA content of the eggs and thus the transcriptomic profile of unfertilized eggs coming from F1 and F7+ populations. In total, 358 genes presented a significant difference of expression between populations.

The GO analysis revealed one biological process term overrepresented among the DEG list that we analyzed, suggesting that this function may be affected by the domestication process. This biological process corresponds to the “immune system” grouping proteins usually involved in the regulation of the adaptative immune response such as activation and proliferation of effective T cells and cytokine production [62], controlling inflammation. However, these genes can be expressed in several tissues and may thus be involved in other biological functions. In fish, the T cell receptor signaling pathway seems to be as complex as in mammals but remains yet to be understood [63,64]. Some transcriptomic studies in eggs, embryos and early larvae showed that genes related with the immune system are often differentially expressed depending on egg quality or the domestication level. Concerning studies on the egg quality, genes representing the immune system are mostly down-regulated in eggs presenting lower potential to properly develop [65,66]. Concerning domestication comparisons, several genes involved in the immune system were down-regulated in a domesticated population of Atlantic salmon (*S*. *salar*) compared to their wild counterparts [15,16]. In the latter case, the populations used were selected for their growth abilities and authors proposed the existence of a trade-off between growth and immune response during the selection process. In another study performed in the steelhead trout (*O*. *mykiss*) [3], authors proposed that it could simply reflect an adaptation of individuals to the captive environment, implying an up-regulation of genes involved in this function in domesticated fish. However, no information on the direction of perturbations (up- or down-regulation of genes in domesticated or wild populations) was given. In our study, the term “immune system” involved genes that were up-regulated either in the F1 population (five genes) or in the F7+ population (four genes). Additionally, most of the highlighted genes code for Butyrophilin proteins involved in several biological functions, on top of their role in the immune system. More particularly, they regulate the oil droplets secretion in the milk produced by mammals [67]. Eurasian perch eggs contain a large oil droplet necessary for proper embryonic development since impairments in its formation have been correlated to embryogenesis defects [68]. It would be interesting to further investigate a potential role of these proteins in the formation of oil droplets in fish eggs.

In addition, some of the genes presenting the wider variations of expression between populations are known to be involved during the early embryogenesis and may partly explain developmental defects leading to early lethalities observed in the F7+ population. The *period2* gene (*per2*) codes for a protein belonging to the basic-helix-loop-helix-PAS (bHLH-PAS) protein family and belongs to the clock genes controlling many developmental and physiological events [69]. However, in several mammalian species, numerous clock genes, including *per2*, have been observed in the developing oocytes and eggs as maternal mRNA. These mRNA are not controlled by circadian rhythms and disappear after the MZT [70,71]. Authors proposed that *per2* could be involved in the control of meiosis but, up to now, no proof has corroborated this hypothesis and further investigations are needed to study their role in this tissue. Similar data have been observed in the developing spermatogonia in mice in which clock genes are expressed without any link with the circadian rhythm. In this study, authors made the hypothesis that this gene is mainly linked to the differentiation process [72]. Our study shows for the first time that a *per2* gene is expressed in fish eggs, suggesting similarities with mammals. In addition, it is highly expressed in eggs of the F1 population. Our study does not allow making any hypothesis about the role of *per2* or if it is dependent or independent of the circadian rhythm. However, it suggests that *per2* role during the gonadogenesis may be evolutionary conserved.

The gene *hace1* codes for the HECT domain and Ankyrin repeat Containing E3 ubiquitin-protein ligase 1. It was first identified as a potential tumor suppressor in humans [73]. Later, absence or mutation of this gene was related with developmental issues such as some neurodevelopmental syndromes in humans [74], a shortening of the body axis, an inhibition of eye pigments formation and a delay in neural tube closure in xenopus [75]. In this last study, authors compared their data with another work performed with zebrafish and showing no clear phenotype in this species. They argued that the zebrafish study revealed the role of HACE1 by using splice-sites morpholino efficient to repress zygotic but not maternal RNA [76] while their study involved translational blocking morpholinos blocking both maternal and zygotic RNA. Thus they suggest that phenotypes in xenopus could be linked to the maternal pool of RNA. Recently, a study demonstrated that HACE1 is involved in the normal development and proper function of the heart in zebrafish [77]. However, authors used splice-site morpholinos suggesting that this cardiac phenotype could be due to later zygotic expression of HACE1. Consequently, even if no maternal role of HACE1 has been studied in the zebrafish, studies performed on xenopus suggest that a maternal expression of these mRNA have consequences on the neural tube development. Up to now, two main targets of Hace1 have been identified: Rac1 involved in the cell cycle control and NADPH oxidase regulating the reactive oxygen species production. A previous study showed that some enzymes involved in the control of the oxidative stress present a variation of expression depending on oocytes quality in Eurasian perch [78]. In some marine invertebrate species, a modification of redox homeostasis may help to avoid the polyspermy during fertilization [79]. In our study, the mRNA abundance of *hace1* is higher in the F7+ population, potentially accelerating NADPH oxydase degradation and thus influencing fertilization. With the current knowledge, no transduction pathway could be favored.

Similarly, the mRNA abundance of *mex3b* mRNA was higher in the domesticated population. *Mex3b*, or *muscle excess 3*, codes for an evolutionary conserved RNA-binding protein involved in post-transcriptional regulations [80]. It is associated to proper embryonic development by establishing antero-posterior patterning in *Tribolium* [81], *Caenorhabditis elegans* [82] and *Xenopus laevis* [83]. As a whole, embryonic patterning is regulated by expression and spatial distribution of many transcripts. In xenopus, *mex3b* mRNA presents a long conserved untranslated 3’UTR involved in its auto regulation [83]. In turn, the protein is involved in the mRNA stability and regulates the abundance of several genes, involved in diverse cellular functions [83]. In the present work, the maternal expression of *mex3b* mRNA presents the highest variation of expression level between the two populations (log2FC > 7 in the RT-qPCR). Consequently, the high expression of this transcript in the F7+ eggs may be linked to fine tuning of numerous molecular functions in the embryo and lead to diverse phenotypes.

Finally, a mRNA coding for an *uncharacterized* protein was found to be significantly more expressed in the eggs laid by F7+ females. BLAST analyses against the Uniprot and NCBI databases showed that they are highly homologous to other uncharacterized proteins in other fish species presenting homologies with some domains of ADP ribosylation enzymes. However, these sequences have not been identified yet.

## Conclusions

Our study showed that reproductive performance of Eurasian perch females may be influenced by the domestication process which is probably closely related to the rearing practices potentially leading to several genetic and/or epigenetic modifications. This study revealed that even if the breeders of two Eurasian perch populations (F1 vs. F7+) were reared and induced in the same conditions, the F1 population started to spawn earlier than the F7+ during the spawning season. In other words, it shows that, in our conditions, the domestication process may influence the responsiveness of females to the reproductive environmental stimuli in captive environment.

The F1 population produced eggs having a better potential to develop properly until hatching compared to eggs from the F7+ population. These differences in egg quality may be linked with the important variation in the eggs transcriptomic content between populations. The identification of several genes presenting distinct expression between the two populations could open new paths of investigation to understand their role and mechanism of regulation during embryogenesis and depending on the domestication level.

Finally, the genetic differentiation analysis between studied populations did not allow us to isolate the domestication as the only factor explaining our data. It reinforces the necessity of studying populations presenting a clearer life history to further understand the dynamic of modifications occurring during the domestication process. It is particularly important in the perspective of a growing pressure toward fish farmers and scientists to initiate selection programs for several fish species.

## Supporting information

S1 Fig Study of oogenesis progression. Evolution of (A) gonado somatic index—GSI and plasmatic levels of (B) 17-β-œstradiol—E2, (C) testosterone and (D) Vitellogenin during oogenesis on the populations F1 and F7+. Bars correspond to mean values ± standard error. Asterisks indicate significant differences between populations at p<0.05 using non-parametric Wilcoxon-Mann-Whitney test. Significance levels are represented as follows: *, p = 0.05–0.01.
(TIF)

S1 Table Primer sequences used for real-time PCR experiment of the 7 most expressed genes in the two populations and reference genes used for data normalization. (DOCX)

S2 Table Survivorship at 24, 48, 72 and 120 hours post-fertilization (hpf) and at hatching in F1 and F7+ populations. Columns 2 and 3 correspond to data presented in the Fig 4A. Different letters mean significant differences between populations an ANOVA one-way repeated measures followed by a TukeyHSD (p ≤ 0.05; n = 19 and 13 for F1 and F7+, respectively). Population means ± SEM are presented.
(DOCX)

S3 Table Deformities rates (Dr) and specific rates in various tissues at hatching in F1 and F7+ populations (Ad—Axis, Cd—Cardiac, Yd—Yolk, Md—Mouth, Od–Other, Ed—Eyes). No significant differences have been identified between the two populations using non-parametric Wilcoxon-Mann-Whitney test (p<0.05; n = 23). Population means ± SEM are presented.
(DOCX)

S4 Table Differentially expressed genes list. PhyloFish gene ID, gene description, False Discovery Rate (p-value) and log(2)FC of the 358 differentially expressed genes on eggs from the F7+ and F1 populations.
(XLSX)

S5 Table Differentially expressed genes listed in the same order as they appear in the clustering analysis. PhyloFish gene ID is shown for each gene and the numbers 1–358 correspond to their position in the figure resulting from clustering analysis.
(XLSX)

S6 Table F7+ gene ontology list. PhyloFish gene ID, gene description, Gene Ontology Category, ID and Terms represented in the eggs transcriptome of the F7+ population. GO Categories are: P = Biological Process, F = Molecular Function and C = Cellular Component.
(XLSX)

S7 Table F1 gene ontology list. PhyloFish gene ID, gene description, Gene Ontology Category, ID and Terms represented in the eggs transcriptome of the F1 population. GO Categories are: P = Biological Process, F = Molecular Function and C = Cellular Component.
(XLSX)

S8 Table Genes belonging to the enriched term found in the overrepresentation analysis. PhyloFish gene ID, gene description, Uniprot protein and gene names, Uniprot Acession Number, False Discovery Rate (p-value), Regulation and log(2)FC of the genes represented in enriched function affected in the transcriptome of the eggs belonging to F7+ and F1 population.
(XLSX)

## References

[1] Price EO. Behavioral Aspects of Animal Domestication. Q Rev Biol. 1984;59(1):1–32.

[2] Price EO. Behavioral development in animals undergoing domestication. Appl Anim Behav Sci. 1999 12;65(3):245–71.

[3] Christie MR, Marine ML, Fox SE, French RA, Blouin MS. A single generation of domestication heritably alters the expression of hundreds of genes. Nat Commun. 2016 2 17;7:10676 10.1038/ncomms10676 26883375 PMC4757788

[4] Beaumont C, Roussot O, Marissal-Avry N, Mormede P, Prunet P, Roubertoux P. Génétique et adaptation des animaux d’élevage: introduction. Inra Prod Anim. 2002;15(5):343–8.

[5] Mignon-Grasteau S, Boissy A, Bouix J, Faure J-M, Fisher AD, Hinch GN, et al Genetics of adaptation and domestication in livestock. Livest Prod Sci. 2005 4 1;93(1):3–14.

[6] Vogt G. Facilitation of environmental adaptation and evolution by epigenetic phenotype variation: insights from clonal, invasive, polyploid, and domesticated animals. Environ Epigenetics [Internet]. 2017 3 29 [cited 2019 May 28];3(1). Available from: https://www.ncbi.nlm.nih.gov/pmc/articles/PMC5804542/10.1093/eep/dvx002 PMC5804542 29492304

[7] Luyer JL, Laporte M, Beacham TD, Kaukinen KH, Withler RE, Leong JS, et al Parallel epigenetic modifications induced by hatchery rearing in a Pacific salmon. Proc Natl Acad Sci. 2017 12 5;114(49):12964–9. 10.1073/pnas.1711229114 29162695 PMC5724268

[8] Gavery MR, Nichols KM, Goetz GW, Middleton MA, Swanson P. Characterization of Genetic and Epigenetic Variation in Sperm and Red Blood Cells from Adult Hatchery and Natural-Origin Steelhead, Oncorhynchus mykiss. G3 Genes Genomes Genet. 2018 11 1;8(11):3723–36. 10.1534/g3.118.200458 PMC6222570 30275172

[9] Rodriguez Barreto D, Garcia de Leaniz C, Verspoor E, Sobolewska H, Coulson M, Consuegra S. DNA Methylation Changes in the Sperm of Captive-Reared Fish: A Route to Epigenetic Introgression in Wild Populations. Mol Biol Evol [Internet]. [cited 2019 Jul 26]; Available from: https://academic.oup.com/mbe/advance-article/doi/10.1093/molbev/msz135/5513369 10.1093/molbev/msz135 PMC6759066 31180510

[10] Anastasiadi D, Piferrer F. Epimutations in developmental genes underlie the onset of domestication in farmed European sea bass. Wittkopp P, editor. Mol Biol Evol. 2019 7 10;msz153. 10.1093/molbev/msz153 PMC6759067 31289822

[11] Dobney K, Larson G. Genetics and animal domestication: new windows on an elusive process. J Zool. 2006;269(2):261–71.

[12] Waaij VD, H E. A resource allocation model describing consequences of artificial selection under metabolic stress. J Anim Sci. 2004 4 1;82(4):973–81. 10.2527/2004.824973x 15080316

[13] Farquharson KA, Hogg CJ, Grueber CE. A meta-analysis of birth-origin effects on reproduction in diverse captive environments. Nat Commun. 2018 3 13;9(1):1055 10.1038/s41467-018-03500-9 29535319 PMC5849764

[14] Pelegri F. Maternal factors in zebrafish development. Dev Dyn. 2003 11;228(3):535–54. 10.1002/dvdy.10390 14579391

[15] Bicskei B, Bron JE, Glover KA, Taggart JB. A comparison of gene transcription profiles of domesticated and wild Atlantic salmon (Salmo salar L.) at early life stages, reared under controlled conditions. Bmc Genomics. 2014;15(1):884. 25301270 10.1186/1471-2164-15-884 PMC4210632

[16] Bicskei B, Taggart JB, Glover KA, Bron JE. Comparing the transcriptomes of embryos from domesticated and wild Atlantic salmon (Salmo salar L.) stocks and examining factors that influence heritability of gene expression. Genet Sel Evol [Internet]. 2016 12 [cited 2018 Mar 6];48(1). Available from: http://www.gsejournal.org/content/48/1/20 10.1186/s12711-016-0200-6 PMC4797325 26987528

[17] Yeates SE, Einum S, Fleming IA, Holt WV, Gage MJG. Assessing risks of invasion through gamete performance: farm Atlantic salmon sperm and eggs show equivalence in function, fertility, compatibility and competitiveness to wild Atlantic salmon. Evol Appl. 2014 4 1;7(4):493–505. 10.1111/eva.12148 24822083 PMC4001447

[18] Lanes CFC, Bizuayehu TT, de Oliveira Fernandes JM, Kiron V, Babiak I. Transcriptome of Atlantic Cod (Gadus morhua L.) Early Embryos from Farmed and Wild Broodstocks. Mar Biotechnol. 2013 12;15(6):677–94. 10.1007/s10126-013-9527-y 23887676

[19] Lanes CFC, Bizuayehu TT, Bolla S, Martins C, de Oliveira Fernandes JM, Bianchini A, et al Biochemical composition and performance of Atlantic cod (Gadus morhua L.) eggs and larvae obtained from farmed and wild broodstocks. Aquaculture. 2012 1;324–325:267–75.

[20] Zupa R, Rodríguez C, Mylonas CC, Rosenfeld H, Fakriadis I, Papadaki M, et al Comparative Study of Reproductive Development in Wild and Captive-Reared Greater Amberjack Seriola dumerili (Risso, 1810). Qiu G-F, editor. PLOS ONE. 2017 1 5;12(1):e0169645 10.1371/journal.pone.0169645 28056063 PMC5215828

[21] Khendek A, Alix M, Viot S, Ledoré Y, Rousseau C, Mandiki R, et al How does a domestication process modulate oogenesis and reproduction performance in Eurasian perch? Aquaculture. 2017 4;473:206–14.

[22] Křišt’an J, Stejskal V, Policar T. Comparison of reproduction characteristics and broodstock mortality in farmed and wild eurasian perch (Perca fluviatilis L.) females during spawning season under controlled conditions. Turk J Fish Aquat Sci. 2012;12(2):191–197.

[23] Teletchea F, Fontaine P. Levels of domestication in fish: implications for the sustainable future of aquaculture. Fish Fish. 2014 6 1;15(2):181–95.

[24] Ben Khadher S, Fontaine P, Milla S, Agnèse J-F, Teletchea F. Genetic characterization and relatedness of wild and farmed Eurasian perch (Perca fluviatilis): Possible implications for aquaculture practices. Aquac Rep. 2016 5 1;3:136–46.

[25] Stepien CA, Haponski AE. Taxonomy, Distribution, and Evolution of the Percidae In: Kestemont P, Dabrowski K, Summerfelt RC, editors. Biology and Culture of Percid Fishes [Internet]. Dordrecht: Springer Netherlands; 2015 [cited 2018 Mar 12]. p. 3–60. Available from: http://link.springer.com/10.1007/978-94-017-7227-3_1

[26] Overton JL, Toner D, Policar T, Kucharczyk D. Commercial Production: Factors for Success and Limitations in European Percid Fish Culture In: Kestemont P, Dabrowski K, Summerfelt RC, editors. Biology and Culture of Percid Fishes: Principles and Practices [Internet]. Dordrecht: Springer Netherlands; 2015 [cited 2019 May 29]. p. 881–90. Available from: 10.1007/978-94-017-7227-3_35

[27] Steenfeldt S, Fontaine P, Overton JL, Policar T, Toner D, Falahatkar B, et al Current Status of Eurasian Percid Fishes Aquaculture In: Kestemont P, Dabrowski K, Summerfelt RC, editors. Biology and Culture of Percid Fishes: Principles and Practices [Internet]. Dordrecht: Springer Netherlands; 2015 [cited 2019 May 29]. p. 817–41. Available from: 10.1007/978-94-017-7227-3_32

[28] Fontaine P, Wang N, Hermelink B. Broodstock Management and Control of the Reproductive Cycle In: Kestemont P, Dabrowski K, Summerfelt RC, editors. Biology and Culture of Percid Fishes [Internet]. Springer Netherlands; 2015 [cited 2017 Mar 20]. p. 103–22. Available from: http://link.springer.com.bases-doc.univ-lorraine.fr/chapter/10.1007/978-94-017-7227-3_3

[29] Gillet C, Dubois JP. A survey of the spawning of perch (Perca fluviatilis), pike (Esox lucius), and roach (Rutilus rutilus), using artificial spawning substrates in lakes. Hydrobiologia. 1995;300(1):409–415.

[30] Wang N, Teletchea F, Kestemont P, Milla S, Fontaine P. Photothermal control of the reproductive cycle in temperate fishes: Photothermal control of reproduction. Rev Aquac. 2010 12;2(4):209–22.

[31] Migaud H, Bell G, Cabrita E, McAndrew B, Davie A, Bobe J, et al Gamete quality and broodstock management in temperate fish. Rev Aquac. 2013 5;5:S194–223.

[32] Abdulfatah A, Fontaine P, Kestemont P, Gardeur J-N, Marie M. Effects of photothermal kinetics and amplitude of photoperiod decrease on the induction of the reproduction cycle in female Eurasian perch Perca fluviatilis. Aquaculture. 2011 12;322–323:169–76.

[33] Sulistyo I. Reproductive cycle and plasma levels of sex steroids in female Eurasian perch Perca fluviatilis. Aquat Living Resour. 1998;11(2):101–10.

[34] Schaerlinger B, Żarski D. Evaluation and Improvements of Egg and Larval Quality in Percid Fishes In: Kestemont P, Dabrowski K, Summerfelt RC, editors. Biology and Culture of Percid Fishes [Internet]. Dordrecht: Springer Netherlands; 2015 [cited 2017 Mar 20]. p. 193–223. Available from: http://link.springer.com/10.1007/978-94-017-7227-3_6

[35] Migaud H, Fontaine P, Kestemont P, Wang N, Brun-Bellut J. Influence of photoperiod on the onset of gonadogenesis in Eurasian perch Perca fluviatilis. Aquaculture. 2004 11;241(1–4):561–74.

[36] Ben Ammar I, Teletchea F, Milla S, Ndiaye WN, Ledoré Y, Missaoui H, et al Continuous lighting inhibits the onset of reproductive cycle in pikeperch males and females. Fish Physiol Biochem. 2015 4;41(2):345–56. 10.1007/s10695-014-9987-7 25233876

[37] Gabe M. Techniques histologiques. Paris: Masson; 1968 vi, 1113.

[38] Rinchard J, Kestemont P. Comparative study of reproductive biology in single- and multiple-spawner cyprinid fish. I. Morphological and histological features. J Fish Biol. 1996;49(5):883–94.

[39] Żarski D, Bokor Z, Kotrik L, Urbanyi B, Horváth A, Targońska K, et al A new classification of a preovulatory oocyte maturation stage suitable for the synchronization of ovulation in controlled reproduction of Eurasian perch, Perca fluviatilis L. Reprod Biol. 2011 11 1;11(3):194–209. 10.1016/s1642-431x(12)60066-7 22139334

[40] Żarski D, Horváth á., Kotrik L, Targońska K, Palińska K, Krejszeff S, et al Effect of different activating solutions on the fertilization ability of Eurasian perch, *Perca fluviatilis* L., eggs. J Appl Ichthyol. 2012 12;28(6):967–72.

[41] Alix M, Chardard D, Ledoré Y, Fontaine P, Schaerlinger B. An alternative developmental table to describe non-model fish species embryogenesis: application to the description of the Eurasian perch (Perca fluviatilis L. 1758) development. EvoDevo [Internet]. 2015 12 [cited 2016 Sep 23];6(1). Available from: http://www.evodevojournal.com/content/6/1/39 10.1186/2041-9139-6-3 PMC4683842 26688712

[42] Alix M, Zarski D, Chardard D, Fontaine P, Schaerlinger B. Deformities in newly hatched embryos of Eurasian perch populations originating from two different rearing systems. J Zool. 2017 6;302(2):126–37.

[43] Pasquier J, Cabau C, Nguyen T, Jouanno E, Severac D, Braasch I, et al Gene evolution and gene expression after whole genome duplication in fish: the PhyloFish database. BMC Genomics [Internet]. 2016 12 [cited 2017 Mar 20];17(1). Available from: http://bmcgenomics.biomedcentral.com/articles/10.1186/s12864-016-2709-z 10.1186/s12864-016-2709-z PMC4870732 27189481

[44] Gotz S, Garcia-Gomez JM, Terol J, Williams TD, Nagaraj SH, Nueda MJ, et al High-throughput functional annotation and data mining with the Blast2GO suite. Nucleic Acids Res. 2008 4 15;36(10):3420–35. 10.1093/nar/gkn176 18445632 PMC2425479

[45] Ozerov MY, Ahmad F, Gross R, Pukk L, Kahar S, Kisand V, et al Highly Continuous Genome Assembly of Eurasian Perch (Perca fluviatilis) Using Linked-Read Sequencing. G3 Genes Genomes Genet. 2018 12 1;8(12):3737–43. 10.1534/g3.118.200768 PMC6288837 30355765

[46] Mi H, Muruganujan A, Huang X, Ebert D, Mills C, Guo X, et al Protocol Update for large-scale genome and gene function analysis with the PANTHER classification system (v.14.0). Nat Protoc. 2019 3;14(3):703 10.1038/s41596-019-0128-8 30804569 PMC6519457

[47] Aljanabi S. Universal and rapid salt-extraction of high quality genomic DNA for PCR- based techniques. Nucleic Acids Res. 1997 11 15;25(22):4692–3. 10.1093/nar/25.22.4692 9358185 PMC147078

[48] Ben Khadher S, Agnèse J-F, Milla S, Teletchea F, Fontaine P. Patterns of genetic structure of Eurasian perch (Perca fluviatilis L.) in Lake Geneva at the end of the spawning season. J Gt Lakes Res. 2015 9;41(3):846–52.

[49] Leclerc D, Wirth T, Bernatchez L. Isolation and characterization of microsatellite loci in the yellow perch (*Perca flavescens*), and cross- species amplification within the family Percidae. Mol Ecol. 2000 7;9(7):995–7. 10.1046/j.1365-294x.2000.00939-3.x 10886663

[50] Wirth T, Saint-Laurent R, Bernatchez L. Isolation and characterization of microsatellite loci in the walleye (*Stizostedion vitreum*), and cross-species amplification within the family Percidae. Mol Ecol. 1999 11;8(11):1960–2. 10.1046/j.1365-294x.1999.00778-3.x 10620241

[51] Borer SO, Miller LM, Kapuscinski AR. Microsatellites in walleye *Stizostedion vitreum*. Mol Ecol. 1999;8(2):336–8. 10065550

[52] Li L, Wang HP, Givens C, Czesny S, Brown B. Isolation and characterization of microsatellites in yellow perch (Perca flavescens). Mol Ecol Notes. 2007;7(4):600–3.

[53] Kearse M, Moir R, Wilson A, Stones-Havas S, Cheung M, Sturrock S, et al Geneious Basic: An integrated and extendable desktop software platform for the organization and analysis of sequence data. Bioinformatics. 2012 6 15;28(12):1647–9. 10.1093/bioinformatics/bts199 22543367 PMC3371832

[54] Belkhir K, Borsa P, Chikhi L, Raufaste N, Bonhomme F, Chikli L, et al GENETIX 4.05, logiciel sous Windows TM pour la génétique des populations. Montpellier, France: Laboratoire Génome, Populations, Interactions, CNRS UMR 5000, Université de Montpellier II; 2004.

[55] Cavanaugh JE, Neath AA. The Akaike information criterion: Background, derivation, properties, application, interpretation, and refinements. WIREs Comput Stat. 2019;11(3):e1460.

[56] R Core Team (2017). R: A language and environment for statistical computing. R Foundation for Statistical Computing. [Internet]. Vienna, Austria.; Available from: URL https://www.R-project.org/.

[57] Hedrick PW. Perspective: Highly Variable Loci and Their Interpretation in Evolution and Conservation. Evolution. 1999;53(2):313–8. 10.1111/j.1558-5646.1999.tb03767.x 28565409

[58] Dekens MPS, Pelegri FJ, Maischein H-M, Nüsslein-Volhard C. The maternal-effect gene futile cycle is essential for pronuclear congression and mitotic spindle assembly in the zebrafish zygote. Dev Camb Engl. 2003 9;130(17):3907–16. 10.1242/dev.00606 12874114

[59] McGinnity P, Prodohl P, Ferguson A, Hynes R, Maoileidigh N o., Baker N, et al Fitness reduction and potential extinction of wild populations of Atlantic salmon, Salmo salar, as a result of interactions with escaped farm salmon. Proc R Soc B Biol Sci. 2003 12 7;270(1532):2443–50. 10.1098/rspb.2003.2520 PMC1691531 14667333

[60] Lund I, Steenfeldt SJ, Suhr KI, Hansen BW. A comparison of fatty acid composition and quality aspects of eggs and larvae from cultured and wild broodstock of common sole (*Solea solea* L.). Aquac Nutr. 2008 12;14(6):544–55.

[61] Bicskei B, Taggart JB, Glover KA, Bron JE. Comparing the transcriptomes of embryos from domesticated and wild Atlantic salmon (Salmo salar L.) stocks and examining factors that influence heritability of gene expression. Genet Sel Evol [Internet]. 2016 12 [cited 2018 Mar 6];48(1). Available from: http://www.gsejournal.org/content/48/1/20 10.1186/s12711-016-0200-6 PMC4797325 26987528

[62] Huang Y, Wange RL. T Cell Receptor Signaling: Beyond Complex Complexes. J Biol Chem. 2004 7 9;279(28):28827–30. 10.1074/jbc.R400012200 15084594

[63] Partula S. Surface markers of fish T-cells. Fish Shellfish Immunol. 1999 5 1;9(4):241–57.

[64] Laing KJ, Hansen JD. Fish T cells: Recent advances through genomics. Dev Comp Immunol. 2011 12 1;35(12):1282–95. 10.1016/j.dci.2011.03.004 21414347

[65] Żarski D, Nguyen T, Le Cam A, Montfort J, Dutto G, Vidal MO, et al Transcriptomic Profiling of Egg Quality in Sea Bass (Dicentrarchus labrax) Sheds Light on Genes Involved in Ubiquitination and Translation. Mar Biotechnol. 2017 2;19(1):102–15. 10.1007/s10126-017-9732-1 28181038 PMC5323488

[66] Mommens M, Fernandes JM, Tollefsen KE, Johnston IA, Babiak I. Profiling of the embryonic Atlantic halibut (Hippoglossus hippoglossus L.) transcriptome reveals maternal transcripts as potential markers of embryo quality. BMC Genomics. 2014 9 30;15(1):829. 25269745 10.1186/1471-2164-15-829 PMC4246526

[67] Robenek H, Hofnagel O, Buers I, Lorkowski S, Schnoor M, Robenek MJ, et al Butyrophilin controls milk fat globule secretion. Proc Natl Acad Sci. 2006 7 5;103(27):10385–90. 10.1073/pnas.0600795103 16801554 PMC1502467

[68] Żarski D, Palińska K, Targońska K, Bokor Z, Kotrik L, Krejszeff S, et al Oocyte quality indicators in Eurasian perch, Perca fluviatilis L., during reproduction under controlled conditions. Aquaculture. 2011 3;313(1–4):84–91.

[69] Crews ST. Control of cell lineage-specific development and transcription by bHLH–PAS proteins. Genes Dev. 1998 3 1;12(5):607–20. 10.1101/gad.12.5.607 9499397

[70] Amano T, Tokunaga K, Kakegawa R, Yanagisawa A, Takemoto A, Tatemizo A, et al Expression analysis of circadian genes in oocytes and preimplantation embryos of cattle and rabbits. Anim Reprod Sci. 2010 9;121(3–4):225–35. 10.1016/j.anireprosci.2010.05.020 20619978

[71] Amano T, Matsushita A, Hatanaka Y, Watanabe T, Oishi K, Ishida N, et al Expression and Functional Analyses of Circadian Genes in Mouse Oocytes and Preimplantation Embryos: Cry1 Is Involved in the Meiotic Process Independently of Circadian Clock Regulation1. Biol Reprod. 2009 3 1;80(3):473–83. 10.1095/biolreprod.108.069542 19020302

[72] Alvarez JD, Chen D, Storer E, Sehgal A. Non-cyclic and Developmental Stage-Specific Expression of Circadian Clock Proteins During Murine Spermatogenesis1. Biol Reprod. 2003 7 1;69(1):81–91. 10.1095/biolreprod.102.011833 12606319

[73] Anglesio MS, Evdokimova V, Melnyk N, Zhang L, Fernandez CV, Grundy PE, et al Differential expression of a novel ankyrin containing E3 ubiquitin-protein ligase, Hace1, in sporadic Wilms’ tumor versus normal kidney. Hum Mol Genet. 2004 9 15;13(18):2061–74. 10.1093/hmg/ddh215 15254018

[74] Hollstein R, Parry DA, Nalbach L, Logan CV, Strom TM, Hartill VL, et al HACE1 deficiency causes an autosomal recessive neurodevelopmental syndrome. J Med Genet. 2015 12;52(12):797–803. 10.1136/jmedgenet-2015-103344 26424145 PMC4717446

[75] Iimura A, Yamazaki F, Suzuki T, Endo T, Nishida E, Kusakabe M. The E3 ubiquitin ligase Hace1 is required for early embryonic development in Xenopus laevis. BMC Dev Biol. 2016 9 21;16:31 10.1186/s12861-016-0132-y 27653971 PMC5031333

[76] Daugaard M, Nitsch R, Razaghi B, McDonald L, Jarrar A, Torrino S, et al Hace1 controls ROS generation of vertebrate Rac1-dependent NADPH oxidase complexes. Nat Commun [Internet]. 2013 7 17 [cited 2018 Apr 3];4 Available from: http://www.nature.com/doifinder/10.1038/ncomms3180 10.1038/ncomms3180 PMC3759041 23864022

[77] Razaghi B, Steele SL, Prykhozhij SV, Stoyek MR, Hill JA, Cooper MD, et al hace1 Influences zebrafish cardiac development via ROS‐dependent mechanisms. Dev Dyn. 2018 2 1;247(2):289–303. 10.1002/dvdy.24600 29024245

[78] Castets M-D, Schaerlinger B, Silvestre F, Gardeur J-N, Dieu M, Corbier C, et al Combined analysis of Perca fluviatilis reproductive performance and oocyte proteomic profile. Theriogenology. 2012 7;78(2):432–442.e13. 10.1016/j.theriogenology.2012.02.023 22578620

[79] Schomer B, Epel D. Redox Changes during Fertilization and Maturation of Marine Invertebrate Eggs. Dev Biol. 1998 11;203(1):1–11. 10.1006/dbio.1998.9044 9806768

[80] Buchet-Poyau K, Courchet J, Hir HL, Séraphin B, Scoazec J-Y, Duret L, et al Identification and characterization of human Mex-3 proteins, a novel family of evolutionarily conserved RNA-binding proteins differentially localized to processing bodies. Nucleic Acids Res. 2007 2 1;35(4):1289–300. 10.1093/nar/gkm016 17267406 PMC1851655

[81] Kimelman D, Martin BL. Anterior–posterior patterning in early development: three strategies. Wiley Interdiscip Rev Dev Biol. 2012 3 1;1(2):253–66. 10.1002/wdev.25 23801439 PMC5560123

[82] Draper BW, Mello CC, Bowerman B, Hardin J, Priess JR. MEX-3 Is a KH Domain Protein That Regulates Blastomere Identity in Early C. elegans Embryos. Cell. 1996 10 18;87(2):205–16. 10.1016/s0092-8674(00)81339-2 8861905

[83] Takada H, Kawana T, Ito Y, Kikuno RF, Mamada H, Araki T, et al The RNA-binding protein Mex3b has a fine-tuning system for mRNA regulation in early Xenopus development. Development. 2009 7 15;136(14):2413–22. 10.1242/dev.029165 19542354

