# Supplementary figures and images for: Domestication may affect the maternal mRNA profile in unfertilized eggs, potentially impacting the embryonic development of Eurasian perch (Perca fluviatilis)
Source: PLoS One. 2019 Dec 31;14(12):e0226878. doi: 10.1371/journal.pone.0226878 (PMC6938363; doi:10.1371/journal.pone.0226878)

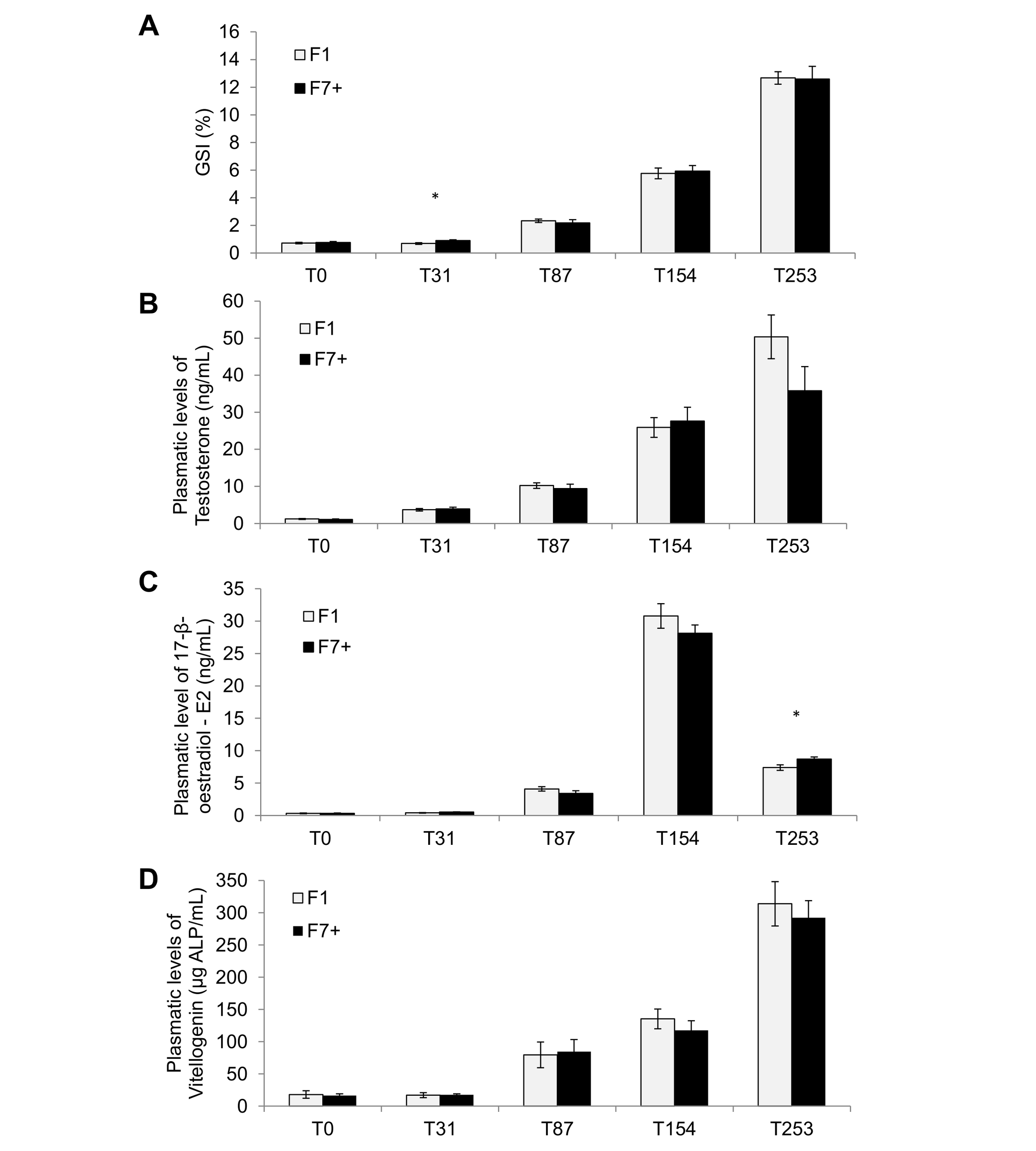

Supplement: S1 Fig — Evolution of (A) gonado somatic index—GSI and plasmatic levels of (B) 17-β-œstradiol—E2, (C) testosterone and (D) Vitellogenin during oogenesis on the populations F1 and F7+. Bars correspond to mean values ± standard error. Asterisks indicate significant differences between populations at p<0.05 using non-parametric Wilcoxon-Mann-Whitney test. Significance levels are represented as follows: *, p = 0.05–0.01. (TIF) [file pone.0226878.s001.tif]
