# Supplementary material for: Domestication may affect the maternal mRNA profile in unfertilized eggs, potentially impacting the embryonic development of Eurasian perch (Perca fluviatilis)
Source: PLoS One. 2019 Dec 31;14(12):e0226878. doi: 10.1371/journal.pone.0226878 (PMC6938363; doi:10.1371/journal.pone.0226878)
Supplement: S1 Table — (DOCX) [file pone.0226878.s002.docx]

S1 Table

| **Target gene** | **Abbreviated name** | **Phylofish/GenBank Gene ID** | **Forward sequence** | **Reverse sequence** | **Annealing temperature** |
| --- | --- | --- | --- | --- | --- |
| ***Higher expressed in F7+ population*** | | | | |  |
| RNA-binding protein MEX3B-like | *mex3b* | MPF_LOC101470955.1.1 | CTCCACGGACTCCTACTTCG | GGCGAGGAGTCAAAAGTCAG | 50°C |
| E3 ubiquitin-protein ligase HACE1 | *hace1* | MPF_HACE1.1.3 | GAACGGACACAAAACCACGG | AGGATCTCACAGGTCTCCCC | 58°C |
| biogenesis of lysosome-related organelles complex 1 subunit 1-like | *bloc1s1* | MPF_LOC100710617.1.3 | ACGCCTGGGCAACTCTAGTA | GCACCCATGCAGTTATGTTG | 58°C |
| - | *uncharacterized protein* | MPF_NEMVEDRAFT_V1G131035.1.1 | GTTTGGTGACAACACCTGGC | CTCGGAGGGGAATTCATGGG | 50°C |
| ***Higher expressed in F1 population*** | | | | |  |
| period circadian protein homolog 2-like | *per* | MPF_LOC101485591.1.2 | AGGGTGGACCGAGTGTACTG | GGGTTAAGGCCGAGGTTTAG | 50°C |
| iodotyrosine dehalogenase 1-like | *iyd* | MPF_LOC100692784.3.3 | TGGTGCTTCGTCTCTGTGTC | GCCTTACTCACCACCACGTT | 58°C |
| Niban-like protein 1 | *nibl1* | MPF_NIBL1.1.1 | GGTTGGGGGTGTAAGACAGA | GTGCTGCATGAAGACAGGAA | 50°C |
| ***Reference genes*** | | | | |  |
| Adenosine kinase-like | *adk* | MPF_LOC101464997.1.1 | CTTCCTGACCGTCTCTTTGG | CCTTGGTCTCGAAGTCTTGC | 50°C |
| ELAV-like protein 1-like | *elavl1* | MPF_LOC100695900.1.2 | GATCGTGAACTACCTGCCCC | TTACCTGCCACTTTGTCCCG | 50°C |
| TATA box-binding protein | *tbp* | MPF_LOC101470168.1.1 | CAGGTGCCAAGGTGAGAGCA | ACAACAGCCCTTGCACAGCA | 55°C |
| 18S ribosomal RNA | - | FJ710875 | CCTGCGGCTTAATTTGACTC | CTCAATCTCGTGTGGCTGAA | 55°C |
