# Supplementary material for: Domestication may affect the maternal mRNA profile in unfertilized eggs, potentially impacting the embryonic development of Eurasian perch (Perca fluviatilis)
Source: PLoS One. 2019 Dec 31;14(12):e0226878. doi: 10.1371/journal.pone.0226878 (PMC6938363; doi:10.1371/journal.pone.0226878)
Supplement: S2 Table — Columns 2 and 3 correspond to data presented in the Fig 4A. Different letters mean significant differences between populations an ANOVA one-way repeated measures followed by a TukeyHSD (p ≤ 0.05; n = 19 and 13 for F1 and F7+, respectively). Population means ± SEM are presented. (DOCX) [file pone.0226878.s003.docx]

S2 Table

|  | Survival based on the total number of embryos studied (~ 100 embryos) | |
| --- | --- | --- |
|  | Populations | |
| Time point | F7+ | F1 |
| 24 h | 54 ± 9% ^a^ | 70 ± 8% ^a^ |
| 48 h | 31 ± 7% ^a^ | 57 ± 7% ^b^ |
| 72 h | 31 ± 8% ^a^ | 56 ± 7% ^b^ |
| 120 h | 32 ± 8% ^a^ | 54 ± 7% ^b^ |
| Hatching (about 15 days) | 24 ± 8% ^a^ | 48 ± 7% ^b^ |

Superscripts refers to the comparisons between populations at each timepoint. Different letters mean significant difference (p ≤ 0.05).
