# Supplementary material for: Domestication may affect the maternal mRNA profile in unfertilized eggs, potentially impacting the embryonic development of Eurasian perch (Perca fluviatilis)
Source: PLoS One. 2019 Dec 31;14(12):e0226878. doi: 10.1371/journal.pone.0226878 (PMC6938363; doi:10.1371/journal.pone.0226878)
Supplement: S3 Table — No significant differences have been identified between the two populations using non-parametric Wilcoxon-Mann-Whitney test (p<0.05; n = 23). Population means ± SEM are presented. (DOCX) [file pone.0226878.s004.docx]

S3 Table

|  | Populations | |
| --- | --- | --- |
|  | F7+ | F1 |
| Dr | 6 ± 1% | 7 ± 1% |
| Ad | 61 ± 8% | 76 ± 4% |
| Cd | 54 ± 10% | 39 ± 7% |
| Yd | 43 ± 10% | 40 ± 7% |
| Md | 34 ± 8% | 23 ± 5% |
| Ed | 7 ± 4% | 9 ± 6% |
| Od | 17 ± 8% | 15 ± 4% |
